# riboSeed: leveraging prokaryotic genomic architecture to assemble across ribosomal regions

**DOI:** 10.1093/nar/gky212

**Published:** 2018-03-28

**Authors:** Nicholas R Waters, Florence Abram, Fiona Brennan, Ashleigh Holmes, Leighton Pritchard

**Affiliations:** 1 Microbiology, School of Natural Sciences, National University of Ireland, Galway, H91 TK33, Ireland; 2 Information and Computational Sciences, James Hutton Institute, Invergowrie, Dundee DD2 5DA, Scotland; 3 Soil and Environmental Microbiology, Environmental Research Centre, Teagasc, Johnstown Castle, Wexford, Y35 TC97, Ireland; 4 Cell and Molecular Sciences, James Hutton Institute, Invergowrie, Dundee DD2 5DA, Scotland

## Abstract

The vast majority of bacterial genome sequencing has been performed using Illumina short reads. Because of the inherent difficulty of resolving repeated regions with short reads alone, only ∼10% of sequencing projects have resulted in a closed genome. The most common repeated regions are those coding for ribosomal operons (rDNAs), which occur in a bacterial genome between 1 and 15 times, and are typically used as sequence markers to classify and identify bacteria. Here, we exploit the genomic context in which rDNAs occur across taxa to improve assembly of these regions relative to *de novo* sequencing by using the conserved nature of rDNAs across taxa and the uniqueness of their flanking regions within a genome. We describe a method to construct targeted pseudocontigs generated by iteratively assembling reads that map to a reference genome’s rDNAs. These pseudocontigs are then used to more accurately assemble the newly sequenced chromosome. We show that this method, implemented as riboSeed, correctly bridges across adjacent contigs in bacterial genome assembly and, when used in conjunction with other genome polishing tools, can assist in closure of a genome.

## INTRODUCTION

**Figure 1.**
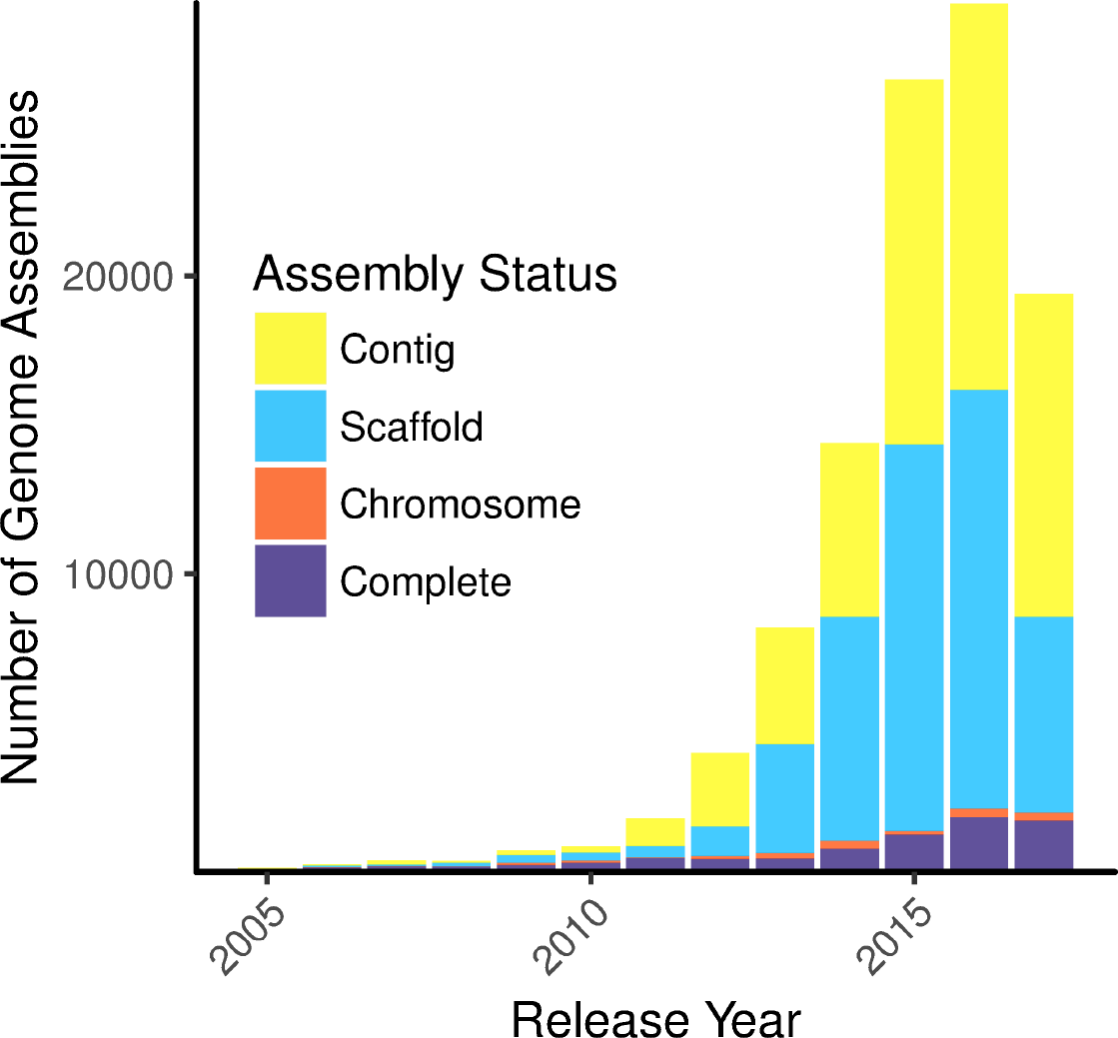
Counts of bacterial assemblies in NCBI Genome database according to completion level by release year; the four levels (Complete, Chromosome, Scaffold and Contig) are ordered from complete to most fragmented (44). Note that Illumina HiSeq was released in 2010. Accessed 14 September 2017.

Sequencing bacterial genomes has become much more cost effective and convenient, but the number of complete, closed bacterial genomes remains a small fraction of the total number sequenced (Figure 1). Even with the advent of new technologies for long-read sequencing and improvements to short read platforms, assemblies typically remain in draft status due to the computational bottleneck of genome closure (1,2). Although draft genomes are often of very high quality and suited for many types of analysis, researchers must choose between working with these draft genomes (and the inherent potential loss of data), or spending time and resources polishing the genome with some combination of *in silico* tools, polymerase chain reaction (PCR), optical mapping, re-sequencing or hybrid sequencing (1,3). Many *in silico* genome finishing tools are available, and we summarize several of these in Table 1.

**Table 1.** Some of the available *in silico* genome polishing tools for gap closure

Tool	Method summary
GapFiller (45)	iteratively uses paired-end reads to close contig junctions
GapCloser (46)	uses paired-end reads to close contig junctions
IMAGE (47)	iteratively uses local assemblies of reads belonging to assembly gaps
CloG (48)	uses trimmed *de novo* contigs in hybrid assembly followed by a stitching algorithm
FGap (49,50)	uses BLAST to find potential gap closures from alternate assemblies, libraries or references.
GFinisher (50)	uses GC-skew to refine assemblies
GapFiller (51)	produces ‘long-reads’ from paired-end sequencing data using a local assembler, which can then be used in a *de novo* assembly
CONTIGuator (52)	uses contigs from a *de novo* assembly along with one or more reference sequences to generate a contig map and PCR primer sets to validate in the lab
Konnector (53)	uses paired-end reads to make long reads to be used in a Bloom filter representation of a de Bruijn graph
MapRepeat(54)	uses a directed scaffolding method to fill in rDNA gaps, but limited to Ion Torrent reads and affected by inversions between rDNAs (40)
Pilon (55)	compares mapping files to an assembly to correct mistakes and fill gaps
GRabB (16)	selectively assembles tandem rDNAs and mitochondria

The Illumina entries in NCBI’s Sequence Read Archive (SRA) (4) outnumber all other technologies combined by about an order of magnitude (Supplementary Table S1). Draft assemblies from these datasets have systematic problems common to short read datasets, including gaps in the scaffolds due to the difficulty of resolving assemblies of repeated regions (5,6). By resolving repeated regions during the assembly process, it may be possible to improve existing assemblies, and therefore obtain additional sequence information from existing short read datasets in the SRA or the European Nucleotide Archive.

The most common repeated regions are those coding for ribosomal RNA operons (rDNAs). As ribosomes are essential for cell function, sequencing of the 16S ribosomal region is widely used to identify prokaryotes and explore microbial community dynamics (7–10). This region is conserved within taxa, yet retains enough variability to act as a bacterial ‘fingerprint’ to separate clades informatively. However, the 16S, 23S and 5S ribosomal subunit coding regions are often present in multiple copies within a single prokaryotic genome, and commonly exhibit polymorphism (11–14). These long, inexactly repeated regions (15) are problematic for short-read genome assembly. As rDNAs are frequently used as a sequence marker for taxonomic classification, resolving their copy number and sequence diversity from short read collections where the assembled genome has collapsed several repeats into a single region could help improve reference databases, increasing the accuracy of community analysis. We present here an *in silico* method, riboSeed, that capitalizes on the genomic conservation of rDNA and flanking sequence within a taxon to improve resolution of these difficult regions and provide a means to benefit from unexploited information in the SRA/ENA short read archives.

riboSeed is most similar in concept to GRabB, the method of Brankovics *et al*. (16) for assembling mitochondrial and rDNA regions in eukaryotes. Both use targeted assembly, but GRabB does not make inferences about the number of rDNA clusters present in the genome or take advantage of their genomic context. In riboSeed, genomic context is resolved by exploiting both the rDNA sequences and their flanking regions, harnessing unique characteristics of the broader rDNA region within a single genome to improve assembly.

The riboSeed algorithm proceeds from two observations: first, that although repeated rRNA coding sequences within a single genome are nearly identical, their flanking regions (i.e. the neighboring locations within the genome) are distinct in that genome, and second, that the genomic contexts of equivalent rDNA sequences are also conserved within a taxonomic grouping (Supplementary Figure S4). riboSeed uses only reads that map to rDNA regions from a reference genome, and is not affected by chromosomal rearrangements that occur outside the flanking regions immediately adjacent to each rRNA.

Briefly, riboSeed uses rDNA regions from a closely related organism’s genome to help generate rDNA cluster-specific ‘pseudocontigs’ derived only from the input short reads, that are seeded into the raw short reads to generate a final assembly. We refer to this process in this work as *de fere novo* (meaning ‘starting from almost nothing’) assembly.

## MATERIALS AND METHODS

**Figure 2.**
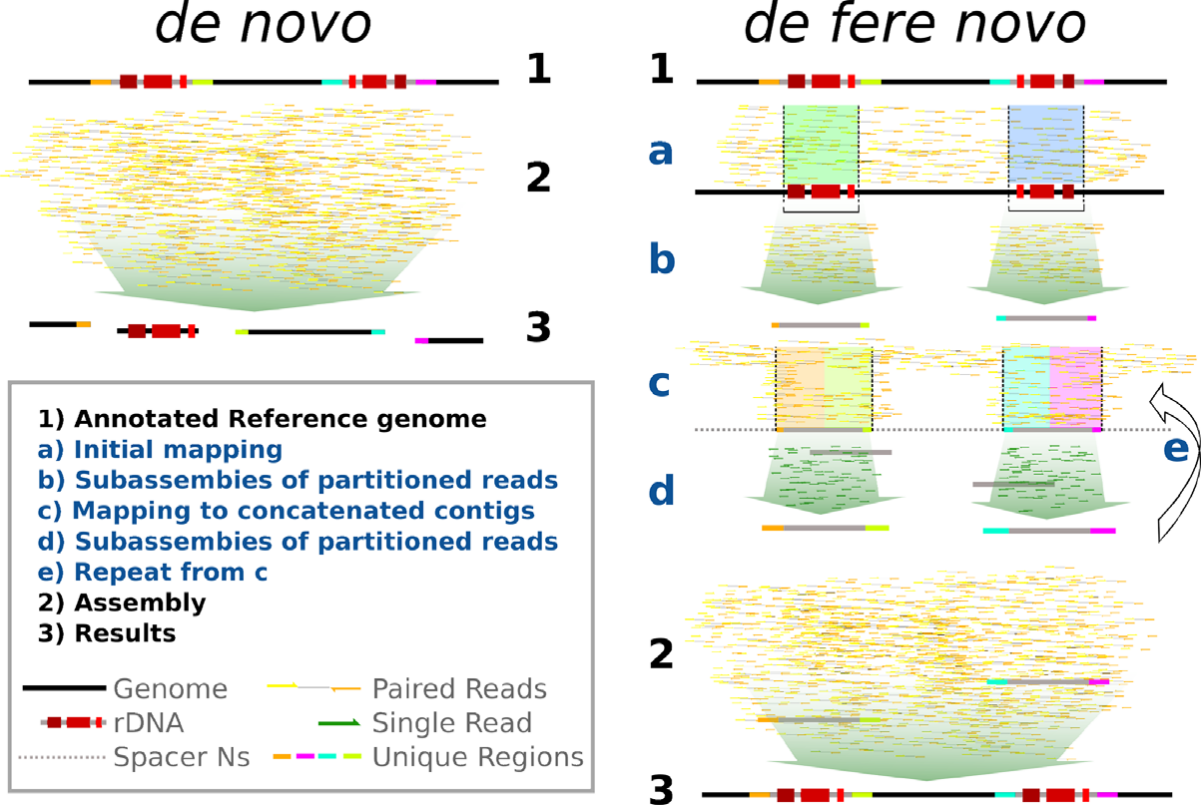
A comparison of *de novo* assembly to *de fere novo* assembly, as implemented in riboSeed. In riboSeed, reads are mapped to a reference genome, and those reads that align to rDNA and flanking regions are extracted. A subassembly for each group of reads that maps to an rDNA region is constructed to produce a ‘pseudocontig’ for each region. These pseudocontigs are concatenated together separated by 1 kb of Ns as a spacer. Reads are then iteratively mapped to the concatenated pseudocontigs, extracted and again subassembled to each region. After the final iteration, the pseudocontigs are included with raw reads in a standard *de novo* assembly. The subassemblies attempt to bridge proper rDNA regions by ensuring that flanking regions (represented here by colors) remain correctly paired. The *de novo* assembly collapses the rDNAs, but *de fere novo* places the rDNAs in the proper genomic context.

We present riboSeed: a software suite that allows users to perform *de fere novo* assembly, given a reference genome sequence from a closely related organism and single or paired-end short reads to be assembled (Figure 2). The code is primarily written in Python3, with accessory shell and R scripts.

riboSeed relies on a closed reference genome assembly that is sufficiently closely related to the isolate being assembled (distance can be estimated using an alignment-free approach such as the KGCAK database (17), or a kmer based method such as Kraken (18)), in which rDNA regions are assembled and assumed to be in the correct genomic context.

In an ideal scenario, reference selection would consist of two steps: isolate identification (using Kraken), and then average nucleotide identity analysis to find the closest complete reference. We outline protocols for reference selection in Supplementary Data, and in the riboSeed documentation at http://riboseed.readthedocs.io/en/latest/REFERENCE.html.

### Usage

Installation (via either conda, pip or GitHub), installs the ribo program. Installation using conda also installs third-party tool dependencies, such as SPAdes, and is recommended. The riboSeed pipeline can be executed with a single command, ribo run or under stepwise control by the user by means of distinct commands. ribo run performs re-annotation of rDNAs in the reference genome (scan command), operon inference (select command) and *de fere novo* assembly (seed command). The most commonly used parameters are accessible via the run command. Alternatively, the full set of parameters for riboSeed can be defined within a configuration file.

All steps in the assembly are controlled by riboSeed commands described below, as ribo <command>:

#### Pre-processing

##### scan

scan uses Barrnap (https://github.com/tseemann/barrnap) to annotate rRNAs in the reference genome, and EMBOSS’s seqret (19) to create GenBank, FASTA and GFF formatted versions of the reference genome. This pre-processing step unifies the annotation vocabulary for downstream processes.

##### select

select infers ribosomal operon structure from the genomic location of constituent 16S, 23S and 5S sequences. Jenks Natural Breaks algorithm is used to group rRNA annotations into likely operons on the basis of genomic coordinates, using the number of 16S annotations to set the number of breaks. Output defines individual rDNA clusters and describes component elements in a plain text file. This output can be manually adjusted before assembly if clustering does not reflect the known arrangement of operons, for example based on visualization of the annotations in a genome browser.

#### *De fere novo* assembly

##### seed

seed implements the algorithm described in Supplementary Figure S1. Short reads for the sequenced isolate are mapped to the reference genome using BWA (20). Reads that map to each annotated rDNA and its flanking regions (where the flanking regions consist of 1 kb upstream and 1 kb downstream of the rDNA, by default) are extracted into subsets (one subset per cluster). Each subset is independently assembled into a representative pseudocontig with SPAdes (21), using the reference rDNA regions as a trusted contig (or untrusted, if mapping quality is poor). Resulting pseudocontigs are evaluated for inclusion in future mapping/subassembly iterations based on length, and concatenated into a pseudogenome in which pseudocontigs are separated by 1 kb of Ns as a spacer. As we are only concerned with flanking regions, the order in which the pseudocontigs are concatenated is arbitrary. A 1 kb spacer length was chosen for this study to ensure that reads did not span the spacer. Pseudocontig generation is iterated at this stage of the algorithm, using the previous round’s pseudogenome as the reference.

After a specified number of iterations (three by default), SPAdes is used to assemble all short reads in a hybrid assembly using pseudocontigs from the final iteration as ‘trusted contigs’ (or ‘untrusted contigs’ if the mapping quality of reads to that pseudocontig falls below a threshold). As a control, the short reads are also *de novo* assembled without the pseudocontigs.

This implementation of riboSeed uses SPAdes to perform both subassembly and the final *de fere novo* assembly, but the pseudocontigs could be submitted to any hybrid assembler that accepts short read libraries and contigs. After assembly, *de fere novo* and *de novo* assemblies are assessed with QUAST (22).

#### Assessment and visualization

##### score

score extracts the regions flanking rDNAs in the reference and in assemblies generated by riboSeed. Flanking regions from an assembly are matched with reference flanking regions using BLASTn. Depending on the ordering of the matches, assembled junctions are called as correct, incorrect or ambiguous based on the criteria outlined below.

##### snag

snag is a helper tool to produce diagnostics and visualization of rDNA sequences in the reference genome. Using the clustering generated by select, sequences for the clusters are extracted from the genome, aligned and Shannon entropy (23) plotted with consensus depth for each position in the alignment.

##### swap

We recommend assessing the performance of the riboSeed pipeline visually using Mauve (24,25), Gingr (26) or similar genome assembly visualizer to compare reference, *de novo* and *de fere novo* assemblies. If contigs appear to be incorrectly joined, the offending *de fere novo* contig can be replaced with syntenic contigs from the *de novo* assembly using the swap script.

##### stack

stack uses bedtools (27) and samtools (20) to compare depth of coverage of reads aligning to the reference genome in the rDNA regions to randomly sampled regions elsewhere in the reference genome. stack takes output from scan, and a BAM file of reads that map to the reference. If the number of scan-annotated rDNAs matches the number of rDNAs in the sequenced isolate, the coverage depths within the rDNAs will be similar to the coverage in other locations in the genome. If the coverage of rDNA regions sufficiently exceeds the average coverage elsewhere in the genome, this may indicate that the reference strain has fewer rDNAs than the sequenced isolate. In this case, using an alternative reference genome may produce improved results.

### Choice of parameters

Settings used for analyses in this manuscript are default (except where otherwise noted) as of riboSeed version 0.4.35 (doi:10.5281/zenodo.1037965).

### Validating assembly across rDNA regions

To evaluate performance of *de fere novo* assembly compared to *de novo* assembly methods, we used Mauve to visualize syntenic regions and contig breaks of each riboSeed assembly in relation to the reference genome used to generate pseudocontigs. We categorized each rDNA in an assembly as either correct, unassembled, incorrect or ambiguous, as follows.

An rDNA assembly is classed as ‘correct’ if two criteria are met: (i) the assembly joins two contigs across an rDNA region such that, based on the reference, the flanking regions of the *de fere novo* assembly are syntenous with those of the reference; and (ii) the assembled contig extends at least 90% of the flanking region length. A cluster is defined as ‘unassembled’ if the ends of one or more contigs align within the rDNA or flanking regions (extension across the rDNA region is not achieved). Finally, if two contigs assemble across a rDNA region in a manner that conflicts with the orientation indicated in the reference genome, suggesting misassembly, the rDNA region is classified as ‘incorrect’.

For analyses where manual inspection was intractable (such as repeated simulations), ribo score was used to categorize the rDNA assemblies. In cases where the program could not distinguish between a correct assembly or an incorrect assembly, the rDNA was classed as ‘ambiguous’.

In all cases, SPAdes was used with the same parameters for both *de fere novo* assembly and *de novo* assembly, apart from addition of pseudocontigs in the *de fere novo* assembly.

## RESULTS

### Characteristics of rDNA flanking regions

**Figure 3.**
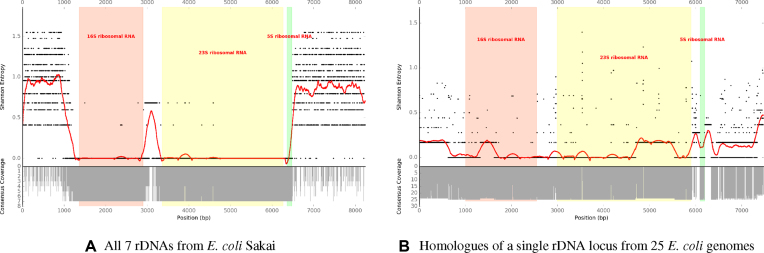
Consensus coverage depth (gray bars) and Shannon entropy (black points, smoothed with a window size of 351 bp as red line) for aligned rDNA regions (16s in red, 23S in yellow, and 5S in green). For the seven *Escherichia coli* Sakai rDNA regions (**A**), entropy sharply increases moving away from the 16S and 5S ends of the operon. In this case flanking regions would be expected to assemble uniquely within a genome. By contrast, the rDNA regions occurring closest to homologous *gmhB* genes from 25 *E. coli* genomes (**B**) show greater conservation in their flanking regions. This indicates that flanking regions are more conserved for homologous rDNA than for paralogous rDNA operons, and implies that related genomes can be useful reference templates for assembling across these regions. Similar plots for each of the GAGE-B genomes used later for benchmarking can be found in Supplementary Figure S4.

The use of rDNA flanking sequences to uniquely identify and place rDNAs in their genomic context requires their flanking sequences to be distinct within the genome for each region. This is expected to be the case for nearly all prokaryotic genomes where rRNA coding sequences are structured as operons. We determined that a 1 kb flanking region was sufficient to include differentiating sequence (Supplementary Figure S2). To demonstrate this, rDNA and 1 kb flanking regions were extracted from *Escherichia coli* Sakai (28) (BA000007.2), a strain in which rDNAs have been well characterized (29). These regions were aligned with MAFFT (30), and consensus depth and Shannon entropy calculated for each position in the alignment (Figure 3A).

Figure 3A and Supplementary Figure S4 show that within a single genome the regions flanking rDNAs are variable between operons. This enables unique placement of reads at the edges of rDNA coding sequences in their genomic context (i.e. there is not likely to be confusion between the placements of rDNA edges within a single genome). In *E. coli* MG1655 (NC_000913.3), the first rDNA is located 363 bases downstream of *gmhB* (locus tag b0200). Homologous rDNA regions were extracted from 25 randomly selected complete *E. coli* chromosomes (Supplementary Table S2). We identified the 20 kb region surrounding *gmhB* in each of these genomes, then annotated and extracted the corresponding rDNA and flanking sequences. These sequences were aligned with MAFFT, and the Shannon entropies and consensus depth plotted (Figure 3B).

Figure 3B shows that equivalent *E. coli* rDNAs, plus their flanking regions, are well-conserved across several related genomes. Assuming that individual rDNAs are monophyletic within a taxonomic group, short reads that can be uniquely placed on a related genome’s rDNA as a reference template are also likely able to be uniquely placed in the appropriate homologous rDNA of the genome to be assembled.

Taken together, when these two properties hold, this allows for unique placement of reads from homologous rDNA regions in the appropriate genomic context. These ‘anchor points’ effectively reduce the number of branching possibilities in de Bruijn graph assembly for each individual rDNA, and thereby permit reconstruction of a complete balanced path through the full rDNA region.

### Simulated reads with artificial chromosome

**Figure 4.**
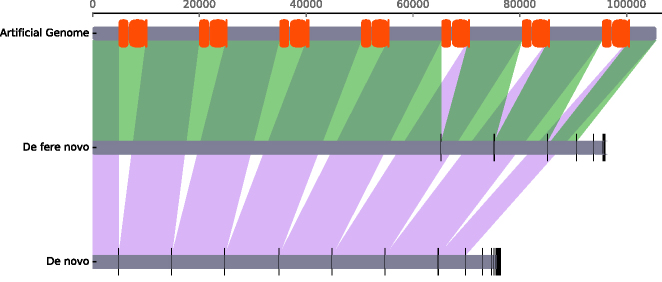
Representative Mauve output describing the results of riboSeed assemblies of simulated reads generated by pIRS from the concatenated *Escherichia coli* Sakai artificial chromosome. Red regions represent rRNA coding sequences, vertical black lines indicate boundaries between assembled contigs and shading represents synteny. From top to bottom: artificial reference chromosome; rDNA clusters (red bars); *de fere novo* assembly and *de novo* assembly (both using *E. coli* MG1655 as the reference). riboSeed’s *de fere novo* method assembles four of seven rDNA regions, but the *de novo* assembly recovers no rDNA regions correctly.

To create a small dataset for testing, we extracted all seven distinct rDNA regions from the *E. coli* Sakai genome (BA000007.2), including 5 kb upstream and downstream flanking sequence, using the tools scan, select and snag. Those regions were concatenated to produce an ∼100 kb artificial test chromosome (see Supplementary Methods). pIRS (31) was used to generate simulated reads (100 bp, 300 bp inserts, stdev 10, 30-fold coverage, built-in error profile) from this test chromosome. These reads were assembled using riboSeed, using the *E. coli* MG1655 genome (NC_000913.3) as a reference. Simulation was repeated eight times to assess variability of method performance on alternative read sets generated from the same source sequence; Figure 4 shows a Mauve alignment for a representative run.

*De fere novo* assembly bridged four of the seven rDNA regions in the artificial chromosome, while *de novo* assembly failed to bridge any (Supplementary Figure S3). To illustrate how choice of reference sequence determines correct assembly through rDNA, we ran riboSeed with the same *E. coli* reads using pseudocontigs derived from the *Klebsiella pneumoniae* HS11286 (CP003200.1) reference genome (32). *De fere novo* assembly with pseudocontigs from *K. pneumoniae* failed, as the reference is too divergent from the reads.

### Effect of reference sequence identity on riboSeed performance

**Figure 5.**
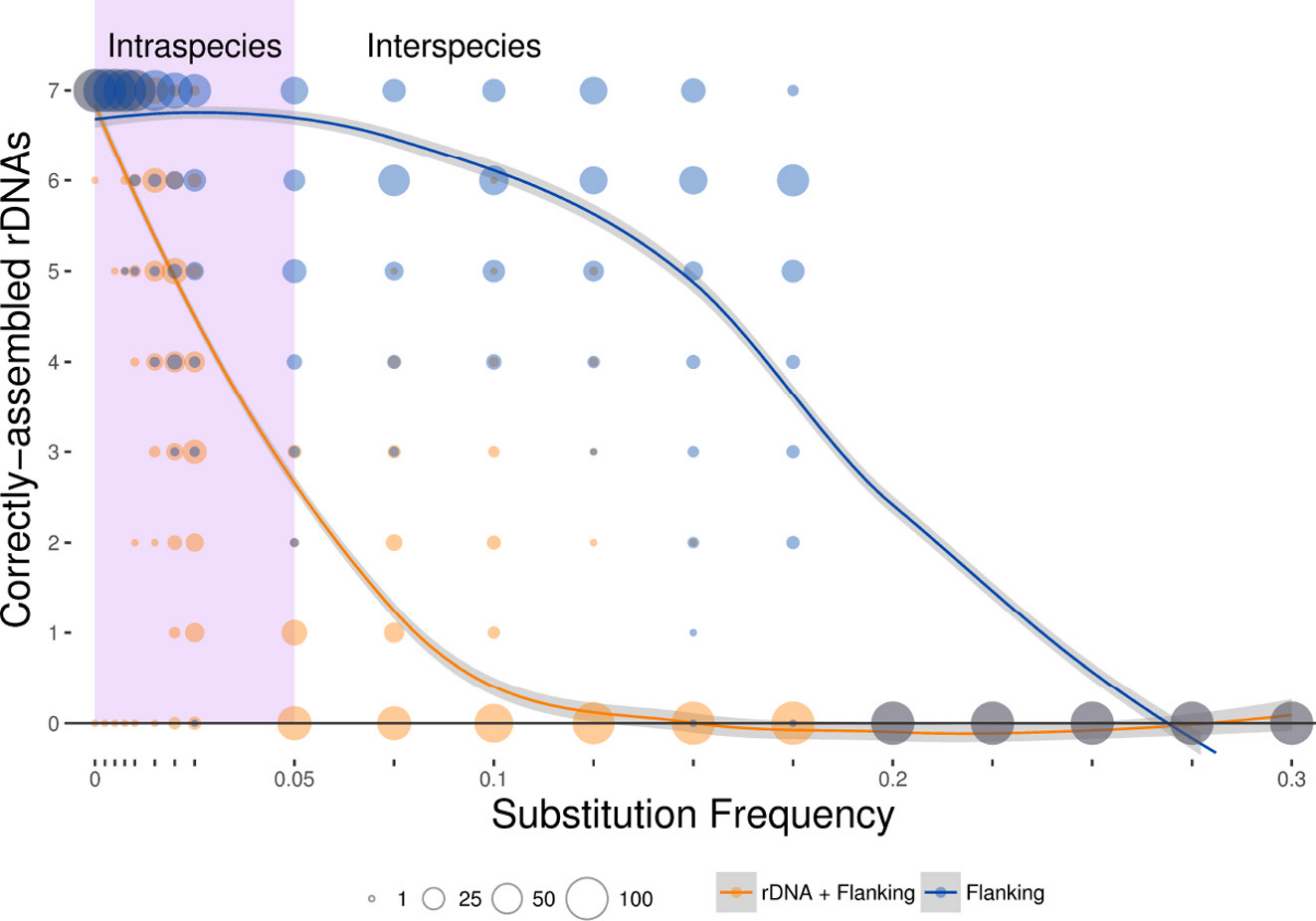
Variants of the artificial chromosome with substitution frequencies between 0 and 0.3 (i.e. up to 300 substitutions per kb). Correctly assembled rDNAs were counted, and the distribution of results shown against the appropriate substitution frequency. Results are shown for models where substitutions are permitted throughout the chromosome (orange), and only in the flanking regions (blue), the latter approximating the relative rate of substitution in rDNA and flanking regions. The lilac area corresponds to substitution frequencies resulting in average sequence identity over 95%, denoting an estimated species boundary. Loess smoothing was used to generate the blue and yellow trendlines. Circle size indicates number of simulations per value. *n* = 100.

To investigate how riboSeed assembly is affected by choice of reference strain, we implemented a simple mutation model to generate reference sequence variants of the artificial chromosome described above, with a specified rate of mutation. A simple model of geometrically distributed mutations at a desired mutation frequency applied across all bases uniformly does not address the disparity of conservation between rDNAs and their flanking region observed in nature, so a second model was applied wherein substitutions are restricted to the rDNA flanking regions. We assembled the artificial chromosome’s reads using the mutated artificial chromosome as a reference, using both models (Figure 5). The maximum substitution rate exceeded our recommended threshold sequence identity, and a corresponding dropoff of performance is observed at a value of 0.2 (corresponding to the 80% mapping percentage identity threshold).

To obtain an estimate of substitution rate for the *E. coli* strains used above, Parsnp (26) and Gingr (26) were used to identify single nucleotide polymorphisms (SNPs) in the 25 genomes used in Figure 3, with respect to the same region in *E. coli* Sakai. An average substitution rate of ∼3.5 substitutions per kb was observed. Compared to the results from simulated genomes, we expect successful riboSeed performance under the model of mutated flanking regions, and partial success under the model of substitutions throughout the region.

Figure 5 indicates that the greater the similarity of the reference sequence to the genome being assembled, the greater the likelihood of correctly assembling all rDNA regions. When mutating only flanking regions (Figure 5), which more closely resembles the relative substitution frequencies of the rDNA regions, the procedure correctly assembles rDNAs with tolerance to substitution frequencies up to ∼30 substitutions per kb. With the widely adopted average nucleotide identity species boundary of 95% (33), we anticipate that riboSeed should correctly place and assemble most rDNA regions when using a complete reference genome of the same species, and that reasonable success will be achieved even when using a more distantly related reference.

### Simulated reads with *E. coli* and *K. pneumoniae* genomes

**Figure 6.**
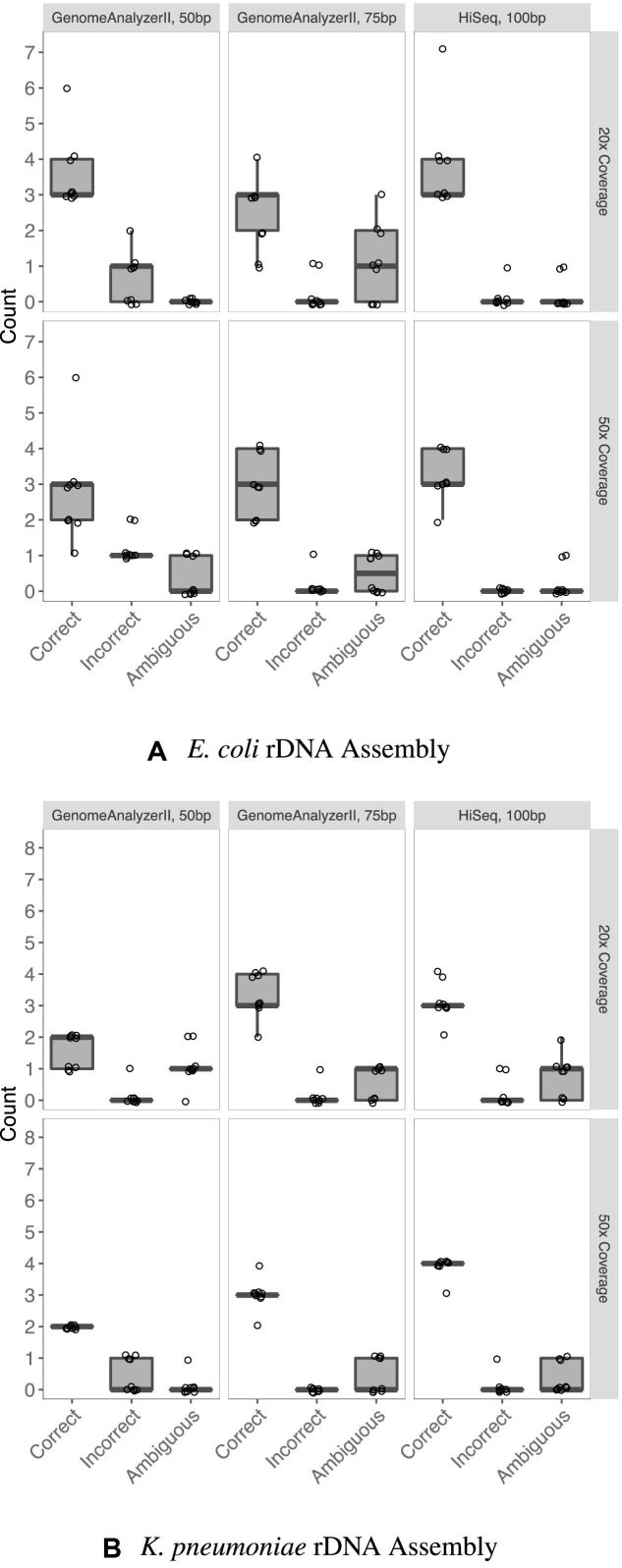
Comparison of *de fere novo* assemblies of simulated reads generated by pIRS. In most cases, increasing coverage depth and read length resulted in fewer misassemblies. Assemblies were scored using score; the *y*-axis reflects the total number of rDNAs in the genome (seven and eight rDNAs, for *Escherichia coli* and *Klebsiella pneumoniae*, respectively). The (*n* = 9) assemblies shown for each genome are the result of differently seeded read simulations.

To investigate the effect of short read length on riboSeed assembly, pIRS (31) was used to generate paired-end reads from the complete *E. coli* MG1655 and *K. pneumoniae* NTUH-K2044 genomes, simulating datasets at a range of read lengths most appropriate to the sequencing technology. In all cases, 300 bp inserted with 10 bp standard deviation and the built-in error profile were used. Coverage was simulated at 20× to emulate low coverage runs and at 50× to emulate coverage close to the optimized values determined by Miyamoto (34) and Desai (35). *De fere novo* assembly was performed with riboSeed using *E. coli* Sakai and *K. pneumoniae* HS11286 as references, respectively, and the results were scored with score (Figure 6).

At either 20× or 50× coverage, *de novo* assembly was unable to resolve any rDNAs with any of the simulated read sets. *De fere novo* assembly with riboSeed showed improvement to both the *E. coli* and *K. pneumoniae* assemblies. Increasing depth of coverage and read length improves rDNA assemblies.

### Benchmarking against hybrid sequencing and assembly

To establish whether riboSeed performs as well with short reads obtained by sequencing a complete prokaryotic chromosome as with simulated reads, we attempted to assemble short reads from a published hybrid Illumina/PacBio sequencing project. The hybrid assembly using long reads was able to resolve rDNAs directly, and provides a benchmark against which to assess riboSeed performance in terms of: (i) bridging sequence correctly across rDNAs, and (ii) assembling rDNA sequence accurately within each cluster.

Sanjar *et al*. published the genome sequence of *Pseudomonas aeruginosa* BAMCPA07-48 (CP015377.1) (36), assembled from two libraries: ca. 270 bp fragmented genomic DNA with 100 bp paired-end reads sequenced on an Illumina HiSeq 4000 (SRR3500543), and long reads from PacBio RS II. The authors obtained a closed genome sequence by hybrid assembly. We ran the riboSeed pipeline on only the HiSeq dataset in order to compare *de fere novo* assembly to the hybrid assembly and *de novo* assembly of the same reads, using the related genome *P. aeruginosa* ATCC 15692 (NZ_CP017149.1) as a reference.

*De fere novo* assembly correctly assembled across all four rDNA regions, whereas *de novo* assembly failed to assemble any rDNA region (Table 3A).

Comparing the BAMCPA07-48 reference to the *de fere novo* assembly, we found a total of nine SNPs in the rDNA flanking regions (Table 2). The same regions from the ATCC 15692 reference used in the *de fere novo* assembly showed 108 SNPs compared to the BAMCPA07-48 isolate. This demonstrates that this subassembly scheme successfully recovers the correct sequence with remarkably few SNPs, despite a large number of differences between the reference and the sequenced isolate, and that the riboSeed method does not simply transpose the reference genome rDNA sequence into the new assembly.

**Table 2.** rDNA region SNPs between hybrid assembly of *P. aeruginosa* BAMCPA07-48 and *de fere novo* assembly in rDNA regions, including 1 kb upstream and downstream of the rDNA

rDNA region (5S,16S, 23S with flanking region)	CP015377.1 Location	Substitution
398001–405418	402331	T → C
	402332	C → T
	404332	C → T
	404380	G → T
1039539–1045687	-	-
1862045–1869194	1864462	A → G
	1868402	A → C
	1868426	A → T
2809154–2816303	2811180	G → A
	2813886	T → C

Further, to assess how riboSeed’s assembly would compare to supplying the whole reference as a trusted contig in SPAdes (a strategy not recommended by the SPAdes authors), we assembled the same reads with the *P. aeruginosa* ATCC 15692 as a trusted contig and compared results to *de fere novo* and *de novo* assemblies. *De novo* assembly yielded the lowest error rates, and *reference-based* assembly yielded the longest contigs, but *de fere novo* assembly exhibited very low error rates, the highest genome recovery fraction and the lowest number of contigs (Supplementary Table S5).

We find that the *de fere novo* assembly using short reads performs better than *de novo* assembly using short reads alone. Comparison of *de fere novo* to hybrid assembly allows assessment of *de fere novo* accuracy, and indicates that *de fere novo* can recover rDNA sequences correctly placed in their genomic context, with a low error rate.

### Case Study: closing the assembly of *S. aureus* UAMS-1

*Staphylococcus aureus* UAMS-1 is a well-characterized, USA200 lineage, methicillin-sensitive strain isolated from an osteomyelitis patient. The published genome was sequenced using Illumina MiSeq (300 bp reads), and the assembly refined with GapFiller as part of the BugBuilder pipeline http://www.imperial.ac.uk/bioinformatics-data-science-group/resources/software/bugbuilder/. Currently, the genome assembly is represented by two scaffolds (JTJK00000000), with several repeated regions acknowledged in the annotations (37). As the rDNA regions were not fully characterized in the annotations, we proposed that *de fere novo* assembly might resolve some of the problematic regions.

Using the same reference *S. aureus* MRSA252 (38) (BX571856.1) with riboSeed as was used in the original assembly, *de fere novo* assembly correctly bridged gaps corresponding to three of the five rDNAs in the reference genome (Table 3B). Furthermore, *de fere novo* assembly bridged two contigs that were syntenic with the ends of the scaffolds in the published assembly, indicating that the regions resolved by riboSeed could improve closure of the genome.

**Table 3.** Comparison of *de novo* and riboSeed’s *de fere novo* assemblies

	Sequenced Strain Name	Platform	Length	Depth	Reference Strain		*de novo*			*de fere novo*		
					Name	rDNAs	**$\checkmark$**	–	×	**$\checkmark$**	–	×
**A**.	*Pseudomonas aeruginosa* BAMCPA07-48	HiSeq	100	200	ATCC 15692	4	**0**	4	0	**4**	0	0
**B**.	*Staphylococcus aureus* UAMS-1	MiSeq	300	110	MRSA252	5	**0**	5	0	**2**	3	0
	*Aeromonas hydrophila* SSU	HiSeq	101	250	ATCC 7966	10	**0**	10	0	**4**	6	0
	*Bacillus cereus* ATCC 10987	MiSeq	250	100	NC7401	14	**0**	14	0	**12**	2	0
	*Bacteroides fragilis* HMW 615	HiSeq	101	250	638R	6	**0**	5	1	**0**	3	3
**C**.	*Rhodobacter sphaeroides* 2.4.1	HiSeq	101	210	ATCC 17029	4	**0**	4	0	**1**	3	0
	*Rhodobacter sphaeroides* 2.4.1	MiSeq	251	100	ATCC 17029	4	**1**	2	1	**1**	2	1
	*Staphylococcus aureus* M0927	HiSeq	101	250	USA300_TCH1516	5	**0**	5	0	**3**	2	0
	*Vibrio cholerae* CO 0132(5)	HiSeq	100	110	El Tor str. N16961	8	**0**	8	0	**5**	3	0
	*Vibrio cholerae* CO 0132(5)	MiSeq	250	100	El Tor str. N16961	8	**0**	8	0	**4**	4	0
	*Xanthomonas axonopodis* pv. *Manihotis* UA323	HiSeq	101	250	pv. *Citrumelo*	2	**0**	1	1	**2**	0	0

✓ correct assembly; – unassembled; × incorrect assembly.

We modified the BugBuilder pipeline (https://github.com/nickp60/BugBuilder) used in the published assembly to incorporate pseudocontigs from riboSeed. Further, we compared the performance of Pilon, GapFiller and no finishing software with both the *de fere novo* and *de novo* assemblies (see Supplementary Table S6). All assemblies resulted in a single scaffold (updates to BugBuilder and many of its dependencies prevented exact recapitulation of the published assembly), but scaffolds varied in length, number of ambiguous bases and resolution of rDNA repeats. In all cases, riboSeed’s *de fere novo* assemblies resulted in more rDNA regions being resolved. In this case, riboSeed was able to assist in bringing an existing high-quality scaffold to near closure.

### Benchmarking against GAGE-B datasets

We used the Genome Assembly Gold-standard Evaluation for Bacteria (GAGE-B) datasets (39) to assess the performance of riboSeed against a set of well-characterized assemblies. These datasets represent a broad range of challenges; low GC content and tandem rDNA repeats prove challenging to the riboSeed procedure.

*Mycobacterium abscessus* has only a single rDNA operon and does not suffer from the issue of rDNA repeats, so it was excluded from this analysis. We also excluded the *Bacillus cereus* VD118 HiSeq dataset, as metagenomic analysis revealed likely contamination (see Supplementary Figure S5 and Supplementary Data).

When the reference used in the GAGE-B study was also the sequenced strain (e.g. *Rhodobacter sphaeroides* and *B. cereus*), we chose an alternate reference, as using the original reference could provide an unfair advantage to riboSeed. The GAGE-B datasets include both raw and trimmed reads; in all cases, the trimmed reads were used. Results are shown in Table 3C.

Compared to *de novo* assembly, the *de fere novo* approach improved the majority of assemblies. In the case of the *S. aureus* and *R. sphaeroides* datasets, particular difficulty was encountered for all references tested. In the case of *Bacteroides fragilis*, the entropy plot (Supplementary Figure S4.3) shows that sequence variability on the 5’ end of the operon is much lower within the genome compared to many of the other within-genome figures, possibly contributing to misassembly.

## DISCUSSION

We demonstrate that regions flanking equivalent rDNAs from related strains show a high degree of conservation in related organisms. This allows us to correctly place rDNAs within a newly sequenced isolate, even in the absence of the resolution that would be provided by long read sequencing. Comparing the regions flanking rDNAs within a single genome, we observed that when considering sufficiently large flanking regions, flanking sequences show enough variability to differentiate each instance of the rDNAs. Taken together with the within-taxon homology, this allows inference of the location (i.e. the flanking regions) of rDNAs, and the variability of these flanking regions within a genome enables unique identification of reads likely belonging to a specific cluster.

The extent of sequence similarity between the sequenced isolate and the reference influences *de fere novo* assembly. If fewer than 80% of reads map to the reference, resulting pseudocontigs are treated as ‘untrusted’ contigs by SPAdes to prevent spurious joining of contigs. Figure 5 shows that although one should preferentially use the closest complete reference available for optimal results, the subassembly method is robust against moderate discrepancies between the reference and sequenced isolate’s flanking regions.

Strains possessing a single rDNA (such as *M. abscessus*) do not suffer from repeated region assembly issues. Similarly, the rRNA coding regions in some taxa (such as *Thermus thermophilus* or *Leptospira interrogans*, see Supplementary section ‘Atypical rDNA operon structure’, Supplementary Figures S6 and 7) are not organized into operons. Such genomes do not require correction with riboSeed.

The method of constructing pseudocontigs implemented by riboSeed relies on having a relevant reference sequence, where the rDNA regions act as ‘bait’, fishing for reads that likely map specifically to that region. This makes riboSeed a valuable tool for assembly or reassembly of bacterial or archaeal strains (Supplementary Tables S7 and 8) for which such a reference is available, but application to community ecology where one may be sequencing novel organisms from unsequenced genera will be limited by the requirement for such a reference genome. Although we show this ‘baiting’ method to be an effective way to partition the reads, a more robust method may be to use a probabilistic representation of equivalent rDNA regions for the sequenced taxon. By using a database of sequence profiles (e.g. Hidden Markov Models) from homologous rDNAs in a taxon, the step of choosing a single most appropriate reference might be circumvented. For datasets where the choice of reference determines riboSeed’s effectiveness, a probabilistic approach may improve performance.

Several checks are implemented after subassembly to ensure that resulting pseudocontigs are fit for inclusion in the next round in the next mapping/subassembly iteration or the final *de fere novo* assembly. If a subassembly’s longest contig is >3× the expected pseudocontig length or shorter than 6 kb (a conservative minimum length of a 16S, 23S and 5S operon), this is taken to be a sign of poor parameter choice so the user is warned, and by default no further seedings will occur to avoid spurious assembly. Such an outcome can be indicative of any of several factors: improper clustering of operons; insufficient or extraneous flanking sequence; sub-optimal mapping; inappropriate choice of *k*-mer length for subassembly; inappropriate reference; or other issues. If this occurs, we recommend testing the assembly with different *k*-mers, changing the flanking length or trying alternative reference genomes. Mapping depth of the rDNA regions is also reported for each iteration; a marked decrease in mapping depth may also be indicative of problems.

Many published genome finishing tools and approaches offer improvements when applied to suitable datasets, but none (including the approach presented in this paper) is able in isolation to resolve all bacterial genome assembly issues. One constraint on the performance of riboSeed is the quality of rRNA annotations in reference strains. Although it is impossible to concretely confirm *in silico*, we (and others (40)) have found several reference genomes during the course of this study that we suspect have collapsed rDNA repeats. We recommend using a tool such as 16Stimator (41) or rrnDB (42) to estimate number of 16S (and therefore rDNAs) prior to assembly, or stack to assess mapping depths after running seed.

As riboSeed relies on de Bruijn graph assembly, the results can be affected by assembler parameters. Care should be taken to explore appropriate settings, particularly in regard to read trimming approach, range of *k*-mers and error correction schemes.

One difficulty in determining the accuracy of rDNA counts in reference genome occurs because genome sequences are often released without publishing the reads used to produce the genome assembly. This practice is a major hindrance when attempting to perform coverage-based quality assessment, such as to infer the likelihood of collapsed rDNAs. While data transparency is expected for gene expression studies, that stance has not been universally adopted when publishing whole-genome sequencing results. To ensure the highest quality assemblies from historical data, we strongly recommend that researchers share raw reads.

## CONCLUSION

Demonstration that rDNA flanking regions are conserved across taxa and that flanking regions of sufficient length are distinct within a genome allowed for the development of riboSeed, a *de fere novo* assembly method. riboSeed utilizes rDNA flanking regions to act as barcodes for repeated rDNAs, allowing the assembler to correctly place and orient the rDNA. *De fere novo* assembly can improve the assembly by bridging across ribosomal regions, and, in cases where rDNA repeats would otherwise result in incomplete scaffolding, can result in closure of a draft genome when used in conjunction with existing polishing tools. Although riboSeed is far from a silver bullet to provide perfect assemblies from short read technology, it shows the utility of using genomic reference data and mixed assembly approaches to overcome algorithmic obstacles. This approach to resolving rDNA repeats may allow further insight to be gained from large public repositories of short read sequencing data, such as SRA, and when used in conjunction with other genome finishing techniques, provides an avenue toward genome closure.

## Supplementary Material

Supplementary Data

## Data Availability

The riboSeed pipeline and datasets generated in this study are available on the riboSeed website, https://nickp60.github.io/riboSeed/. The software is released under the MIT licence. The modified BugBuilder pipeline used here is provided at https://github.com/nickp60/BugBuilder. Reference genomes used for this study can be found in Supplementary Table S3, and the versions of other software used in this study are found in Supplementary Table S4.
