# Supplementary material for: riboSeed: leveraging prokaryotic genomic architecture to assemble across ribosomal regions
Source: Nucleic Acids Res. 2018 Mar 28;46(11):e68. doi: 10.1093/nar/gky212 (PMC6009695; doi:10.1093/nar/gky212)
Supplement: Supplementary Data [file gky212_supplemental_files.pdf]

Supplementary Data

riboSeed: leveraging prokaryotic genomic architecture to assemble across ribosomal regions

Nicholas R. Waters,[1,2] Florence Abram,[1] Fiona Brennan,[1,3] Ashleigh Holmes,[4] and Leighton Pritchard[2*]

[1] *Department of Microbiology, School of Natural Sciences, National University of Ireland, Galway, Ireland*
[2] *Information and Computational Sciences, James Hutton Institute, Invergowrie, Dundee DD2 5DA, Scotland*
[3] *Soil and Environmental Microbiology, Environmental Research Centre, Teagasc, Johnstown Castle, Wexford, Ireland*
[4] *Cell and Molecular Sciences, James Hutton Institute, Invergowrie, Dundee DD2 5DA, Scotland*

[*] To whom correspondence should be addressed: leighton.pritchard@hutton.ac.uk

Compiled: 2018/02/13 12:56:00

Extended Methods

Reference Selection Recommendations

Using a recently-diverged reference sequence maximizes chances of a successful assembly. We have outlined two methods to select an appropriate reference for a given isolate: a robust method using Kraken, and a quick method using Reads2Type.

Method 1: Kraken

Kraken [1] is a *k*-mer-based phylogeny tool that can be used to identify the strains present in a metagenomic dataset; installation and usage instructions can be found here: https://ccb.jhu.edu/software/kraken/. After downloading and installing Kraken, along with the MiniKraken database from their website, Kraken can be run on an isolate's reads, generating the a taxonomy report.

The MiniKraken database was built from all the complete genomes from RefSeq, allowing the user to identify which strain in the database has the closest match to the sequenced isolate.

Method 2: Reads2Type and cgFind

Reads2Type [2] is also a *k*-mer-based phylogeny tool, but it relies on a lightweight, prebuilt database of 55-mers from a set of reference strains. This allows the analysis to be performed in the web browser, and it does not require the user to upload complete read files, allowing it to perform well when either speed or network access is limited. It works by taking one read at a time from the input file, generating 55-mers, and comparing to a prebuilt database. If there is not enough resolution information to identify the isolate by that read alone, additional reads are processed until a single taxonomy identification is achieved. This method works best on trimmed reads. Instructions and the webserver can be found at https://cge.cbs.dtu.dk/services/Reads2Type/

Given the genus and species from Reads2Type, users can make use of cgFind, a web tool we developed to provide easy access to downloadable genomes based on the complete prokaryotic genomes found in NCBI. The tool can be found at https://nickp60.github.io/cgfind.

Making the artificial chromosome

The artificial chromosome used for testing was constructed using the `makeToyGenome.sh` script included in the GitHub repository (`https://github.com/nickp60/riboSeed`) under the **`scripts`** directory. Briefly, the 7 rDNA regions from the *E. coli Sakai* genome were extracted with 5kb flanking sequence upstream and downstream; these sequences were then concatenated end to end to form a single, *approx*100kb sequence containing the 7 rDNAs as well as their flanking context.

Effect of reference sequence identity on riboSeed performance

The following range of substitutions were introduced into a artificial genome using the **`runDegenerate.sh`** script (included in the GitHub repository under the **`scripts`** directory), which facilitates the following procedure: 0.0,

0.0025, 0.0050, 0.0075, 0.0100, 0.0150, 0.0200, 0.0250, 0.0500, 0.0750, 0.1000, 0.1250, 0.1500, 0.1750, 0.2000, 0.2250, 0.2500, 0.2750, 0.3000. An artificial test genome is constructed (see above), and reads simulated using pIRS (100bp, 300bp inserts, stdev 10, 30-fold coverage, built-in error profile). Then, for each of a range of substitution frequencies, substitutions are introduced into the simulated genome, either just in the flanking regions or uniformly throughout. riboSeed is run on the reads using the mutated genome as the reference, and the results are evaluated with riboScore. This script was run 100 times, using a different random seed each time. As pseudo random number generation may differ between operating systems, comparable but not identical results can be expected.

Key Parameters

`--ref_as_contig`

The assembly that results from including riboSeed's "long reads" is sensitive to the manner in which they are incorporated into the *de novo* assembly. Here, for our analyses, we used the SPAdes assembler [3], as it has built-in ways to include contigs (using the "–trusted-contigs" or "–untrusted-contigs") in FASTA format. Other assemblers could be used, but most require long reads to have a quality score associated with them, preventing direct use of riboSeed's long reads.

As mentioned in the Methods section, riboSeed uses the reference rDNA region in the initial subassembly; in subsequent subassemblies, the longest contig of the previous subassembly is used. These regions can be treated one of four ways using the `--ref_as_contig` argument: `trusted`, `untrusted`, `infer`, or `ignore`. Additionally, if the user is worried that the reference rDNA will too heavily influence the initial subassembly, they can enable the `--initial_consensus` flag to use a mapping consensus assembly instead of the de Bruijn graph based assembly from SPAdes.

The default manner in which rDNA regions (either from the reference or from the previous iteration's subassembly) behaviour is to infer (`--ref_as_contig infer`): if the percent of reads mapping to the (whole) reference sequence is over 80%, than the rDNA region will be included as a trusted contig. If below 80%, the reads will be treated as untrusted.

If a user wishes to have the subassemblies only using the reads (true *de novo* assembly), they can use the `ignore` option. We only recommend this with very close references.

Further, if the user wishes to explicitly define the behaviour, `trusted` or `untrusted` can be provided to the `--ref_as_contig` argument.

`--score_min`

By default, the accepted alignment score for BWA mapping is $\frac{1}{2}$ the read length. If needed, this can be increased for greater stringency when dealing with more divergent references, or decreased to include more reads, which may be advantageous when assembling a low coverage dataset.

Table S1: Hits resulting from searching the SRA database for various sequencing technologies as of January, 2017

Search term	Hits	Percentage
illumina	2242225	94.27
pacbio	21131	0.89
ion	30560	1.28
roche	42445	1.78
oxford	12301	0.52
solid	29791	1.25
Total	2378453	100

Table S2: Accessions for 25 *E. coli* genomes used to calculate substitution rate

GCA_000005845.2_ASM584v2
GCA_000019385.1_ASM1938v1
GCA_000026245.1_ASM2624v1
GCA_000026345.1_ASM2634v1
GCA_000026545.1_ASM2654v1
GCA_000146735.1_ASM14673v1
GCA_000257275.1_ASM25727v1
GCA_000520055.1_ASM52005v1
GCA_000732965.1_ASM73296v1
GCA_001007915.1_ASM100791v1
GCA_001442495.1_ASM144249v1
GCA_001469815.1_ASM146981v1
GCA_001660565.1_ASM166056v1
GCA_001660585.1_ASM166058v1
GCA_001753565.1_ASM175356v1
GCA_001888075.1_ASM188807v1
GCA_001901025.1_ASM190102v1
GCA_001936315.1_ASM193631v1
GCA_002056065.1_ASM205606v1
GCA_002078295.1_ASM207829v1
GCA_002156825.1_ASM215682v1
GCA_002163935.1_ASM216393v1
GCA_002192275.1_ASM219227v1
GCA_002220265.1_ASM222026v1
GCA_900096795.1_Ecoli_AG100_Sample3_Doxycycline_Assembly

All available at `ftp://ftp.ncbi.nlm.nih.gov/genomes/all/GCA/`

Table S3: Strain names and accessions for reference genomes used in this study.For the full list, which includes strains used in the supplementary data, SRA accession numbers for reads, and more, please consult the supplementary file "strain_metadata.tab"

.

Strain Name	Accession
E. coli MG1655	NC_000913.3
E. coli Sakai	BA000007.2
A. hydrophila ATCC 7966	NC_008570.1
B. cereus ATCC 10987	AE017194.1
B. cereus NC7401	NC_016771.1
B. fragilis 638R	FQ312004.1
K. pneumoniae	CP003200.1
R. sphaeroides ATCC 17029	NC_009049.1, NC_009050.1
S. aureus TCH1516	NC_010079.1
S. aureus MRSA252	BX571856.1
V. cholerae El Tor str. N16961	NC_002505.1, NC_002506.1
X. axonopodis pv. Citrumelo	CP002914.1
P. aeruginosa BAMCPA07-48	CP015377.1
P. aeruginosa ATCC 15692	NZ_CP017149.1

Table S4: Software Versions

Tool	Version
Mauve	2015-02-13 build 0
BLAST+	2.2.28+
Barrnap	0.8
BWA	0.7.8-r455
samtools	1.4.1
MAFFT	v7.310
SPAdes	v3.9.0
QUAST	4.4
bedtools	2.17.0
EMBOSS	6.5.7
pIRS	2.0.2
seqtk	1.2-r94
Parsnp	v1.2

Table S5: QUAST [4] results of *P. aeruginosa* BAMCPA07-48 assemblies comparing *de fere novo* assembly, *de novo* assembly, and reference-based assembly (where the *P. aeruginosa* ATCC 15692 reference is included in the *de novo* assembly as a trusted contig). Blue and red highlight the best and worst results, respectively. riboSeed's *de fere novo* assembly either outperforms or performs comparably to *de novo* assembly in all categories. Using the reference as a trusted contig results in longer assemblies but with a much higher rate of mismatches, indels, and misassemblies.

	de fere novo	***de novo***	**reference-based**
Genome fraction (%)	98.106	97.868	98
Duplication ratio	1.001	1.001	1.017
Largest alignment	630503	402463	757685
Total aligned length	6893293	6876715	6993532
NGA50	176510	176510	135376
LGA50	12	13	14
# misassemblies	2	2	9
Misassembled contigs length	212498	212498	2347560
# mismatches per 100 kbp	1.89	1.69	11.66
# indels per 100 kbp	2.48	2.44	2.94
# N's per 100 kbp	0	0	0
# contigs	154	159	388
Largest contig	630503	402463	1103106
Total length	6893293	6876715	7237564
Total length (>= 1000 bp)	6865091	6848513	7130244
Total length (>= 10000 bp)	6687664	6663031	6617370
Total length (>= 50000 bp)	6242010	6168232	5534330

Table S6: Assembling *S. aureus* UAMS-1 with BugBuilder. We did not have access to critical information about the pipeline parameters used in the original assembly. This prevented exact recapitulation of the published results. Therefore, we approximated the settings based on notes from the publication. The performance of Pilon [5], GapFiller [6], or no finishing software was assessed with both the *de fere novo* and *de novo* assemblies. rDNA counts were visually determined using Mauve; all other metrics were generated with QUAST, using the scaffolds from the assemblies and the *S. aureus* MRSA252 reference. Misassembly/mismatch stats were removed, as the reference and sequenced strain have an average nucleotide identity of 97.62%, and the misassemblies cannot be differentiated from strain differences with QUAST. Blue and red highlight the best and worst results, respectively.

	de fere novo			***de novo***		
	**GapFiller**	**Pilon**	–	**Gapfiller**	**Pilon**	–
rDNAs	3	3	3	0	0	0
# contigs ($\geq$ 0 bp)	1	1	1	1	1	1
Total length	2773352	2781986	2763179	2768273	2770267	2752929
Reference length	2902619	2902619	2902619	2902619	2902619	2902619
GC (%)	32.78	32.80	32.78	32.72	32.73	32.71
Reference GC (%)	32.81	32.81	32.81	32.81	32.81	32.81
Unaligned length	20087	15391	19075	18980	16819	20141
Genome fraction (%)	94.736	94.941	94.508	94.487	94.604	94.105
Duplication ratio	1.001	1.004	1.000	1.002	1.003	1.000
# N's per 100 kbp	151.44	58.59	160.65	184.23	111.00	200.73
Largest alignment	403935	469521	403508	459120	459623	403508
Total aligned length	2753265	2766595	2744104	2749293	2753448	2732788
NA50	223096	195659	222384	205820	164646	222384
NGA50	176550	177158	222384	157488	164646	176039
LA50	5	5	5	5	6	5
LGA50	6	6	5	6	6	6

Performance Across Prokaryotic Phyla

Performance on Archaeal Data

We assessed the effectiveness of riboSeed in assembling archaeal genomes. Most (~55%) archaeal genomes have only a single rDNA, and none has been observed to have more than four. As riboSeed requires a sequencing dataset and a reference genome, our ability to benchmark was limited; of the 104 entries in *rrn*DB with multiple rDNAs, only 7 had multiple entries at the species level. Among those, only 2 had publicly available short read data. We used riboSeed to re-assemble *Methanosarcina barkeri Fusaro DSMZ804* (SRR2064286) and *Methanobacterium formicicum st. JCM10132* (DRR017790).*Methanosarcina barkeri Fusaro DSMZ804* and *Methanobacterium formicicum st. BRM9* were the only isolates that were suitable for riboSeed, in that there was publicly available short read data, more than a single rDNA operon, and an appropriate complete reference genome at the species level. Results are shown in Table S7A.

Methanosarcina barkeri Fusaro DSMZ804 was sequenced using an Illumina HiSeq2000 with 101bp paired-end reads, with an average fragment length of 400bp. Using seqtk (https://github.com/lh3/seqtk), we downsampled to use 5% of the 19.4Gbp dataset. *Methanosarcina barkeri str. Wiesmoor* (CP009526.1) was used as a reference. The resulting riboSeed assembly showed correct assembly of 3 of 3 rDNAs, while *de novo* assemble failed to resolve any.

M. formicicum st. JCM10132 was sequenced on an Ion Torrent PGM, generating 106.5Mbp of 89bp single-end reads. *M formicicum BRM9* (CP006933.1) was used as a reference. While riboSeed with default parameters did not resolve any of the assembly gaps (final assembly *k*-mers 21, 33, 55, and 77), re-running the final assembly with *k*-mers of 21, 33, 55, 77, and 99 resulted in closing 2 of 2 rDNA gaps. We are unsure why the addition of 99-mers improved assembly with 89-bp reads, but we are actively investigating this. This shows that riboSeed is not limitted to Illumina short read data, and can be applied to Ion Torrent data.

Taken together, we show that given appropriate datasets and parameters, archaeal datasets can be processed in the same manner used for bacteria.

Performance Across Bacterial Phyla

In order to assess riboSeed's wider applicability, we selected additional datasets representing major bacterial phyla for those not already present in our analysis. In all cases, riboSeed improved the assemblies compared to the *de novo* with no missassemblies introduced (Table S7B). Thus, we conclude that riboSeed can be applied to a wide range of organisms.

Table S7: Comparison of *de novo* and riboSeed's *de fere novo* assemblies

	Organism	Sequenced Strain Name	SRA	Reference Strain Name	Accession	rDNAs	*de novo* ✓	*de novo* –	*de novo* ×	*de fere novo* ✓	*de fere novo* –	*de fere novo* ×
**A.**	*Methanobacterium formicicum*	JCM10132	DRR017790	BRM9	CP006933.1	3	**0**	3	0	**3**	0	0
	Methanosarcina barkeri	Fusaro DSMZ804	SRR2064286	Wiesmoor	CP009526.1	2	**0**	2	0	**2**	0	0
**B.**	*Corynebacterium diphtheriae*	NCTC 13129	SRR4271515	241	NC_016782.1	5	**0**	5	0	**3**	2	0
	Chlamydia trachomatis	Population 1	SRR5942978	434/Bu	NC_010287.1	2	**0**	2	0	**2**	0	0
	Clostridioides difficile	C00005970	ERR251735	630	AM180355.1	11	**0**	11	0	**9**	2	0
	Burkholderia cepacia	DHQP2016-12-119	SRR6334321	ATCC 25416	NZ_CP012981.1	6	**0**	6	0	**3**	3	0
	Myxococcus xanthus	DSM 16526	SRR4236978	DK 1622	NC_008095.1	4	**0**	4	0	**4**	0	0
	Helicobacter cinaedi	MRY12-0051	DRR090193	ATCC BAA-847	NC_020555.1	3	**0**	3	0	**3**	0	0
	Mycoplasma hominis	Australia	ERR1938252	ATCC 23114	NC_013511.1	2	**0**	2	0	**2**	0	0

✓ correct assembly; – unnassembled; × incorrect assembly

Table S8: Taxa used in this study

**Kingdom**	**Class**	**Order**	**Family**	**Genus**	**Species**
Actinobacteria	Actinobacteria	Corynebacteriales	Corynebacteriaceae	*Corynebacterium*	*diphtheriae*
Bacteroidetes	Bacteroidia	Bacteroidales	Bacteroidaceae	*Bacteroides*	*fragilis*
Chlamydiae	Chlamydiia	Chlamydiales	Chlamydiaceae	*Chlamydia*	*trachomatis*
Firmicutes	Bacilli	Bacilliales	Bacillaceae	*Bacillus*	*cereus*
Firmicutes	Bacilli	Bacilliales	Staphylococcaceae	*Staphylococcus*	*aureus*
Firmicutes	Clostridia	Clostridiales	Peptostreptococcaceae	*Clostridioides*	*difficile*
Proteobacteria	Alphaproteobacteria	Rhodobacterales	Rhodobacteraceae	*Rhodobacter*	*sphaeroides*
Proteobacteria	Betaproteobacteria	Burkholderiales	Burkholderiaceae	*Burkholderia*	*cepacia*
Proteobacteria	Deltaproteobacteria	Myxococcales	Myxococcaceae	*Myxococcus*	*xanthus*
Proteobacteria	Epsilonproteobacteria	Campylobacterales	Helicobacteraceae	*Helicobacter*	*cinaedi*
Proteobacteria	Gammaproteobacteria	Aeromonadales	Aeromonadaceae	*Aeromonas*	*hydrophila*
Proteobacteria	Gammaproteobacteria	Enterobacterales	Enterobacteriaceae	*Escherichia*	*coli*
Proteobacteria	Gammaproteobacteria	Enterobacterales	Enterobacteriaceae	*Klebsiella*	*pneumoniae*
Proteobacteria	Gammaproteobacteria	Pseudomonadaceae	Pseudomonadaceae	*Pseudomonas*	*aeruginosa*
Proteobacteria	Gammaproteobacteria	Vibrionales	Vibrionaceae	*Vibrio*	*cholerae*
Proteobacteria	Gammaproteobacteria	Xanthomonadales	Xanthomonadaceae	*Xanthomonas*	*axonopodis*
Tenericutes	Mollicutes	Mycoplasmatales	Mycoplasmataceae	*Mycoplasma*	*hominis*
Euryarchaeota	Methanomicrobia	Methanosarcinales	Methanosarcinaceae	*Methanosarcina*	*barkeri*
Euryarchaeota	Methanobacteria	Methanobacteriales	Methanobacteriaceae	*Methanobacterium*	*formicicum*

```
riboSeed (reference, riboSelect_clusters, reads, iters,
 flanking_width)
    ref = reference;
    clusters = parse riboSelect_clusters;
    region = clusters + flanking_width;
    for i in iters do
        map reads to ref;
        for cluster in clusters do
            filter and extract reads region;
            subassemble;
            return pseudocontig;
        end
        assess subassembly;
        if success then
            make pseudogenome from pseudocontigs ;
            ref = pseudogenome ;
        end
    end
    run assembler with reads and pseudocontigs;
end
```

Figure S1: Pseudocode of riboSeed algorithm

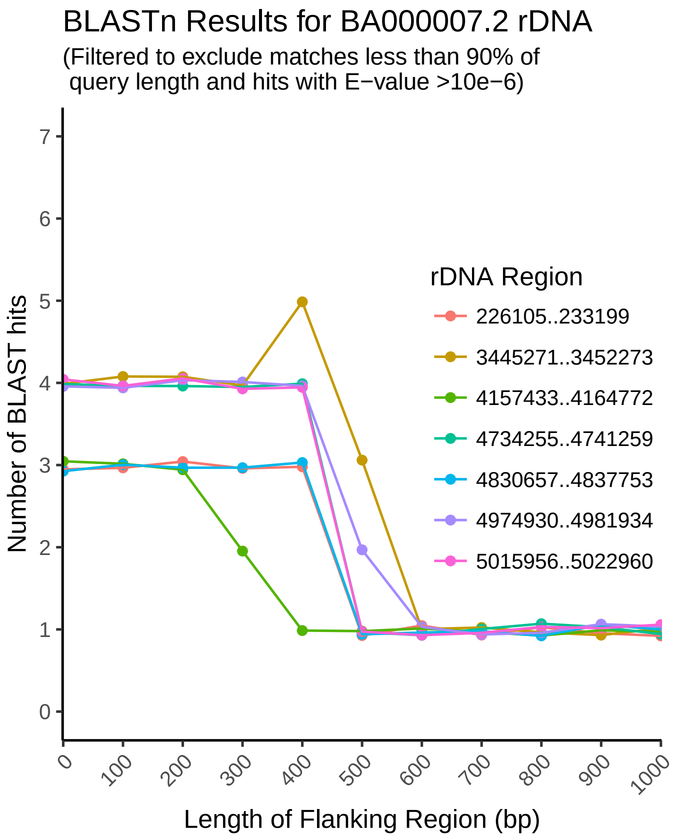

Figure S2: BLASTn was used to perform *in silico* DNA-DNA hybridization of all rDNA regions from *E. coli Sakai* with variable flanking lengths. The number of hits is a proxy for occurrences in the genome; increasing the flanking length increases the specificity. (Points are jittered to aid visibility for overlapping values.)

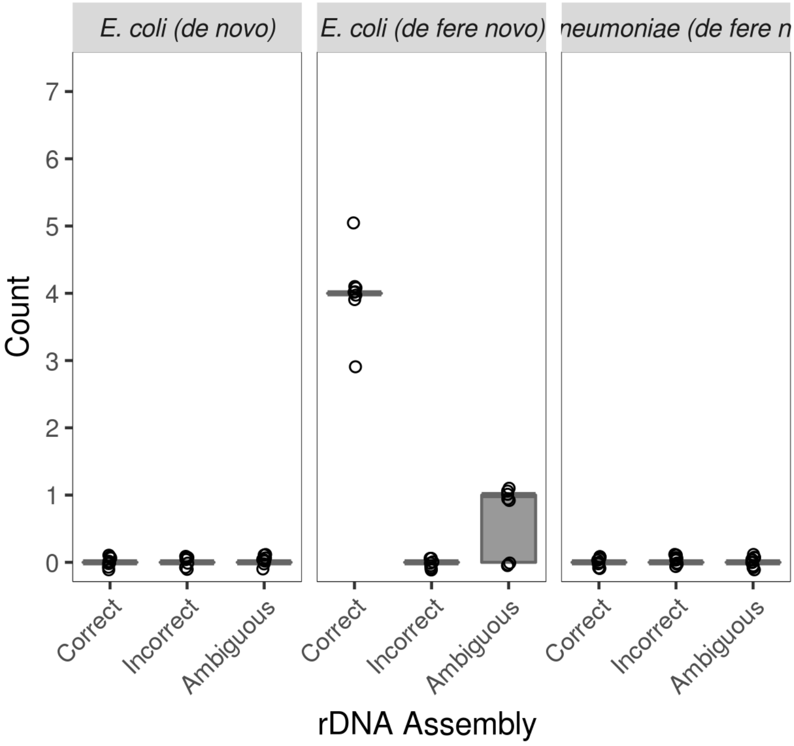

Figure S3: Assembly of artificial genome. *De fere novo* results in closure of 3-5 rDNAs with the correct reference. No rDNAs are correctly assembled using *K. pneumoniae* as the reference, or with *de novo* assembly. Scored with riboScore.py. N=8.

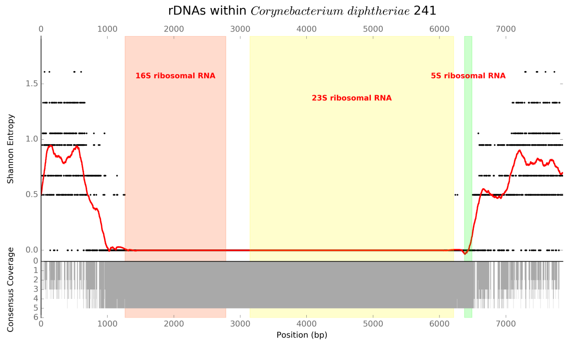

(S4.1)

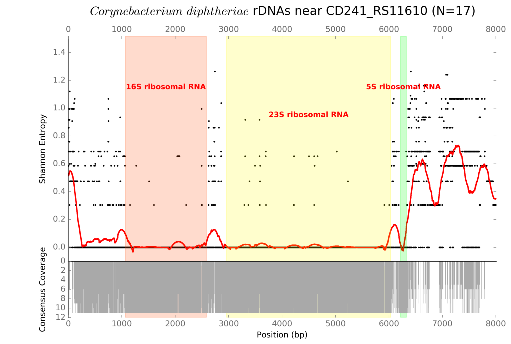

(S4.2)

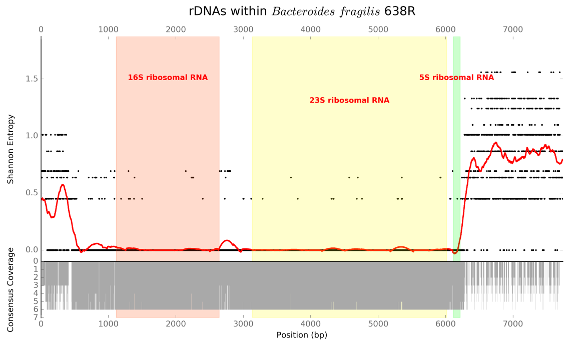

(S4.3)

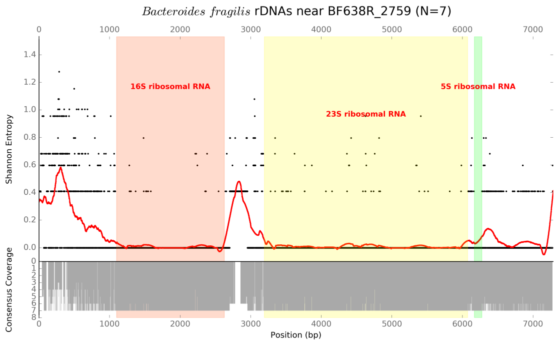

(S4.4)

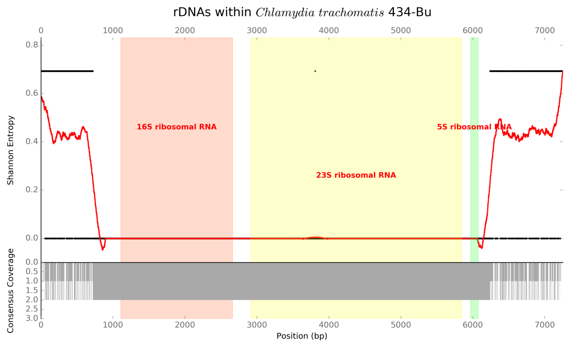

(S4.5)

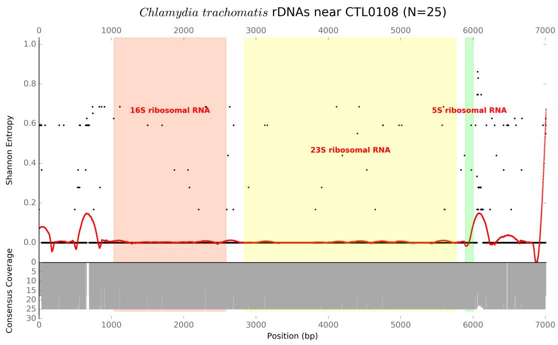

(S4.6)

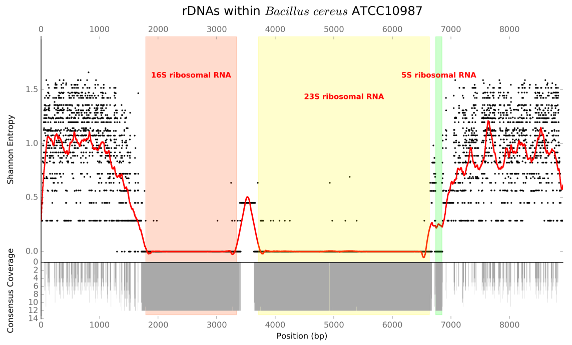

(S4.7)

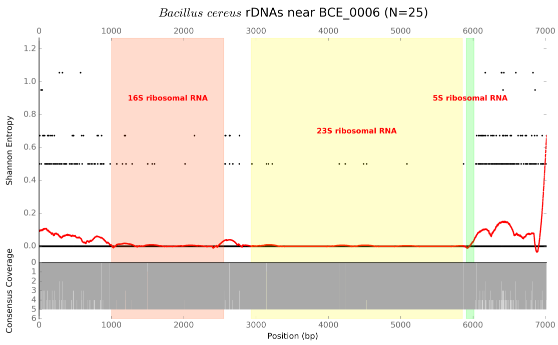

(S4.8)

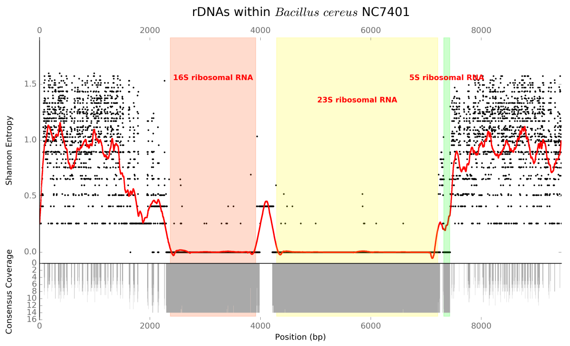

(S4.9)

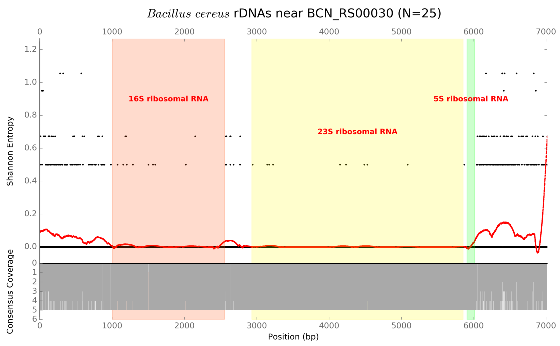

(S4.10)

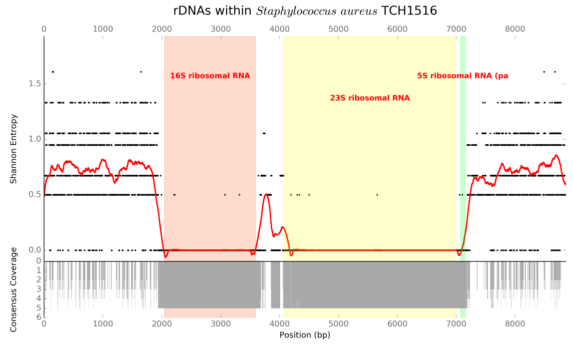

(S4.11)

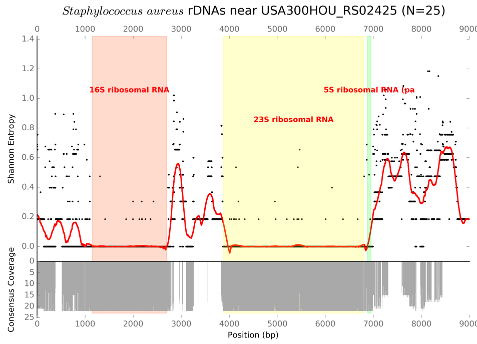

(S4.12)

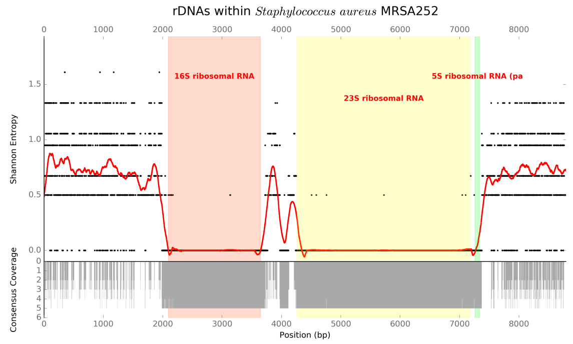

(S4.13)

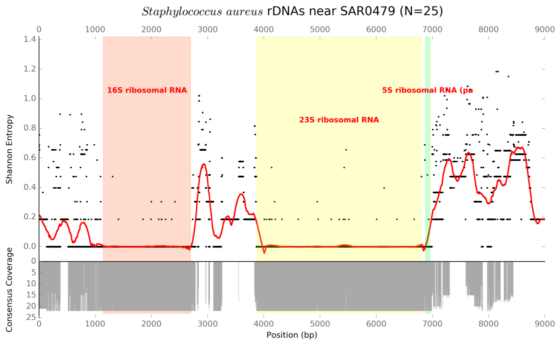

(S4.14)

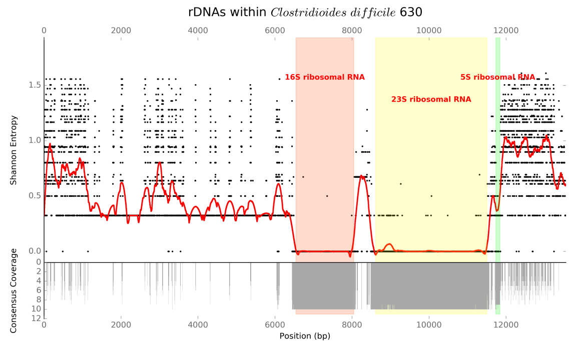

(S4.15)

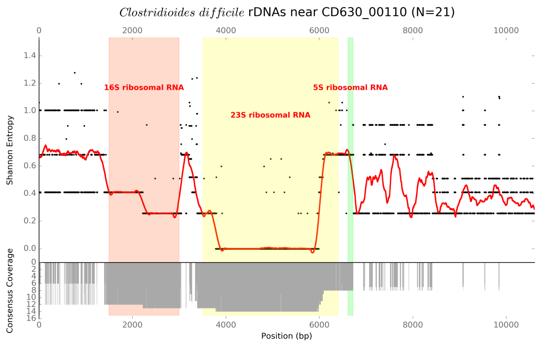

(S4.16)

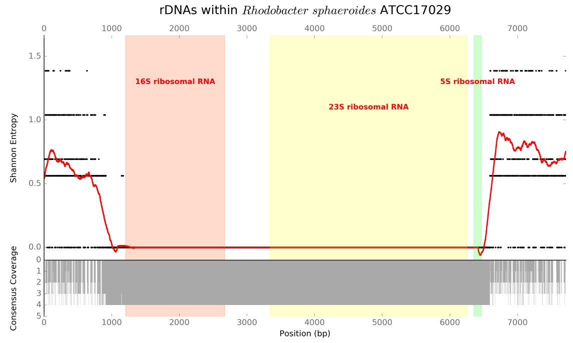

(S4.17)

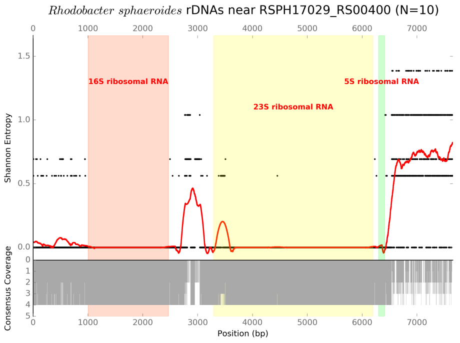

(S4.18)

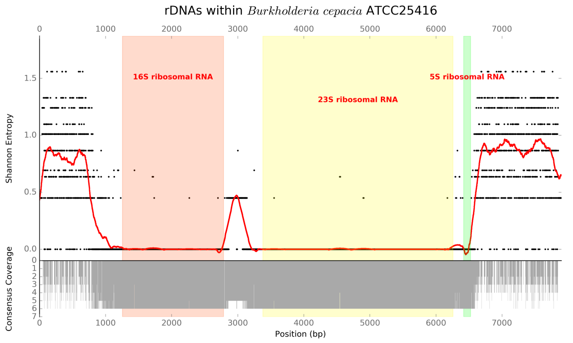

(S4.19)

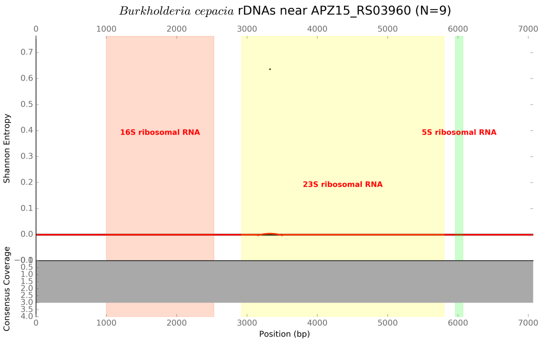

(S4.20)

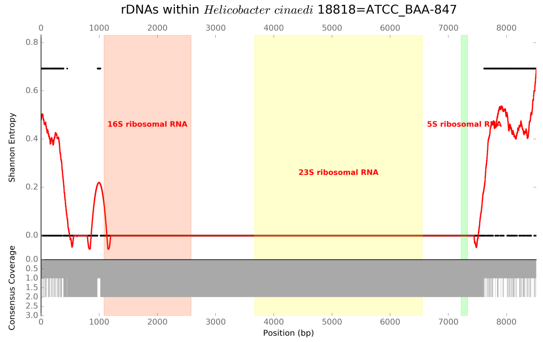

(S4.21)

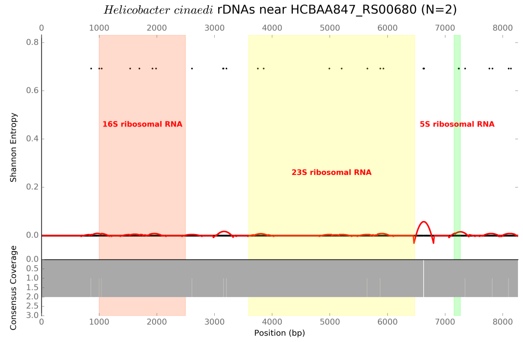

(S4.22)

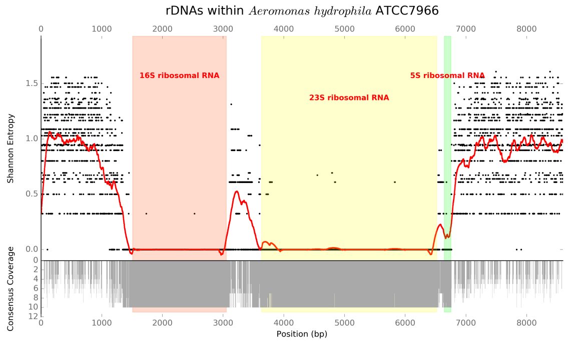

(S4.23)

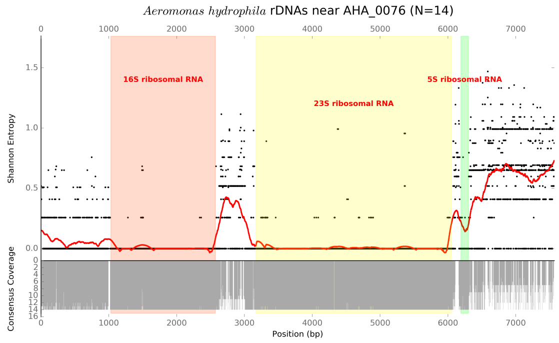

(S4.24)

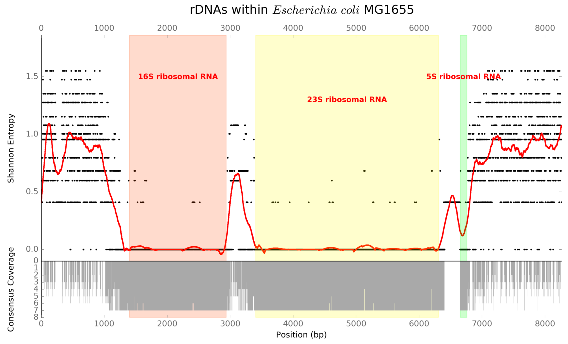

(S4.25)

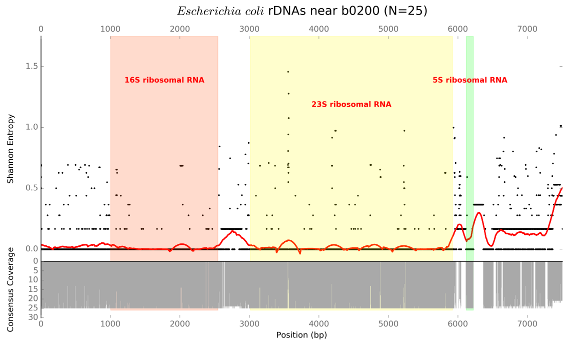

(S4.26)

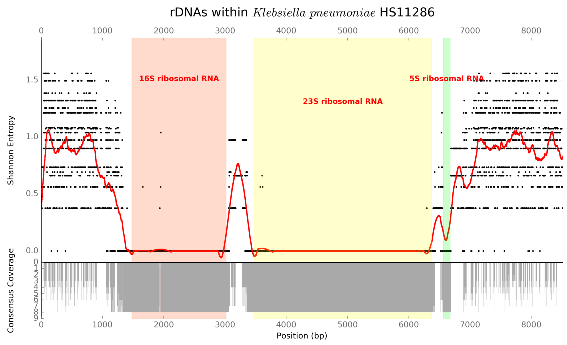

(S4.27)

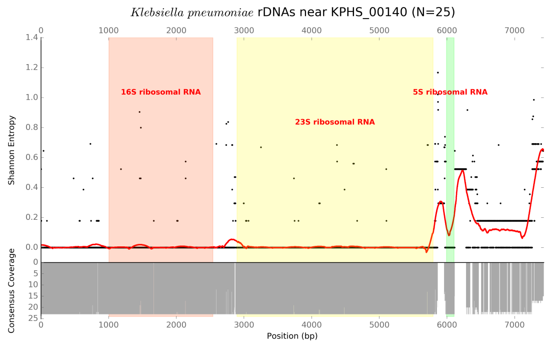

(S4.28)

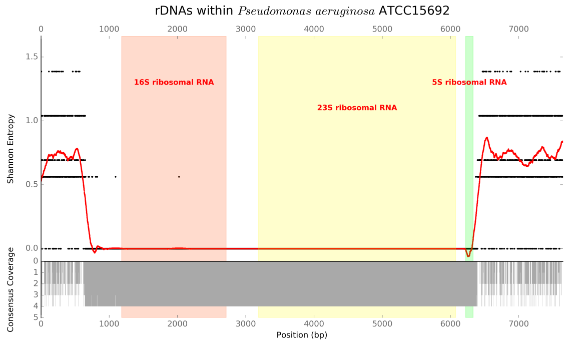

(S4.29)

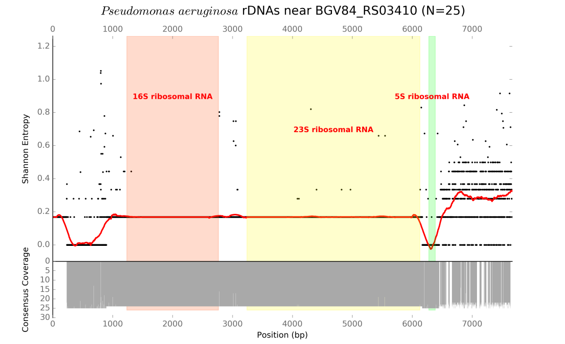

(S4.30)

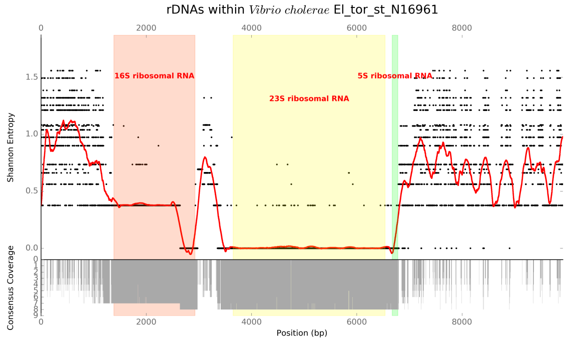

(S4.31)

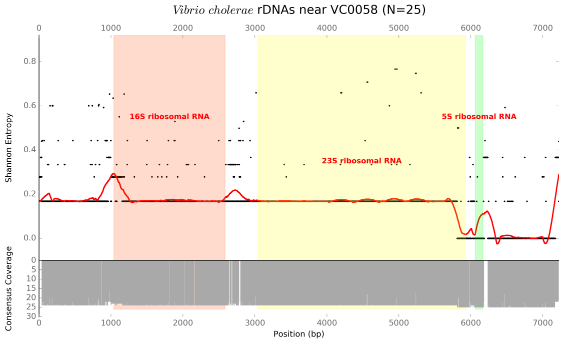

(S4.32)

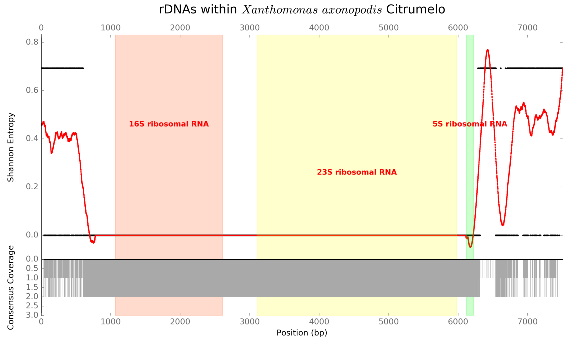

(S4.33)

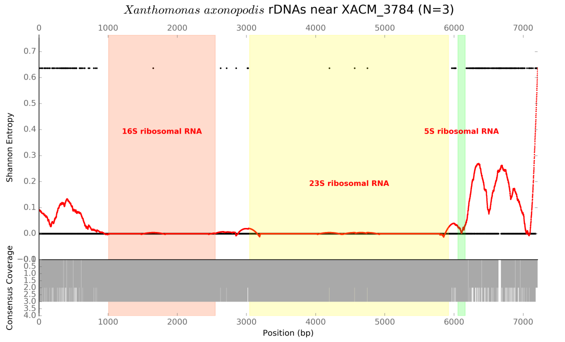

(S4.34)

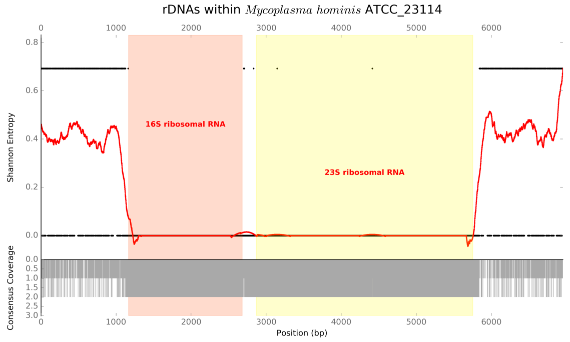

(S4.35)

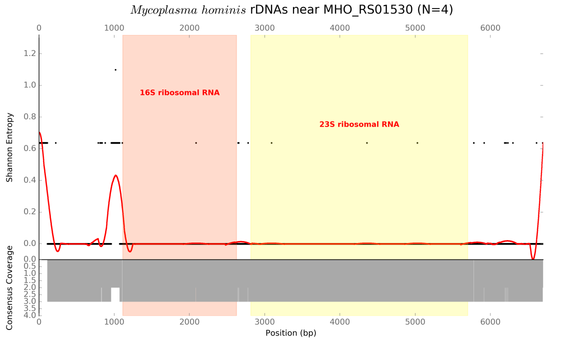

(S4.36)

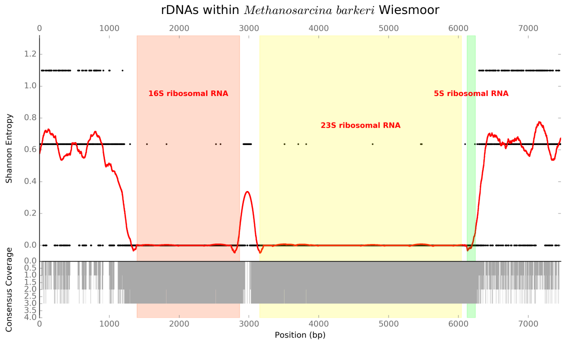

(S4.37)

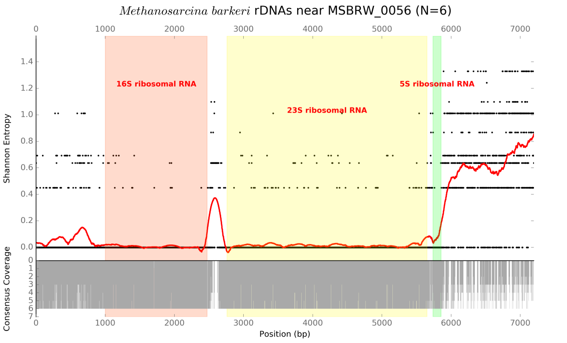

(S4.38)

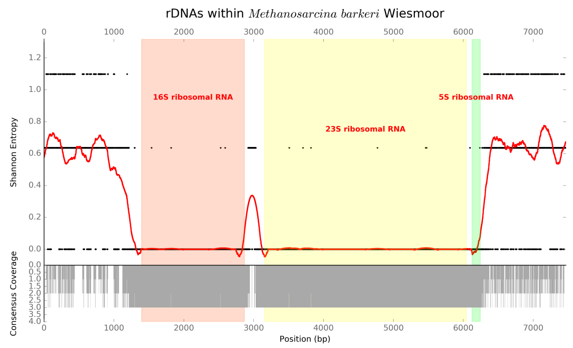

(S4.39)

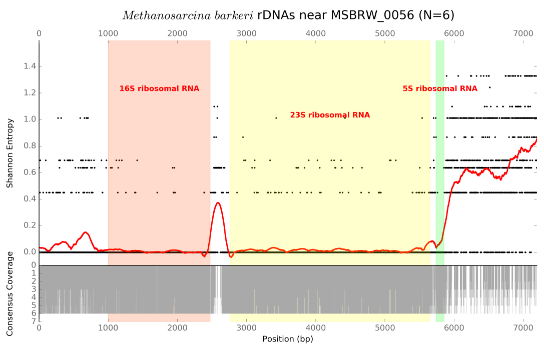

(S4.40)

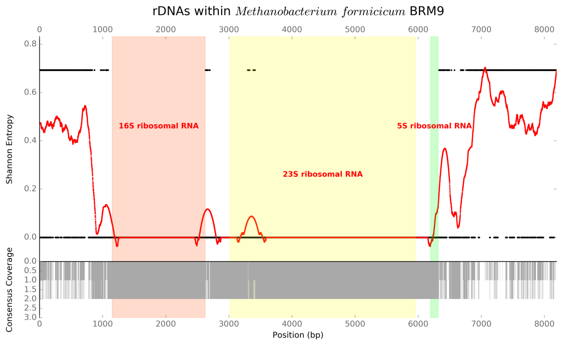

(S4.41)

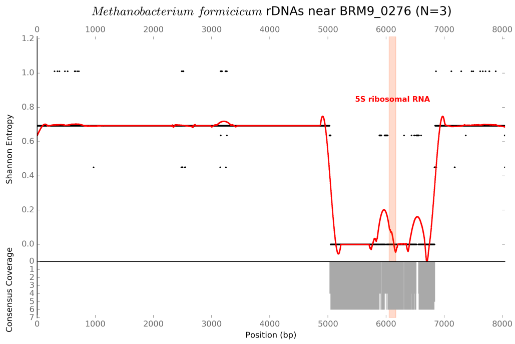

(S4.42)

Figure S4: riboScan.py, riboSelect.py, and riboSnag.py were run on all the genomes used as references for *de fere novo* assemblies. Consensus alignment depth (grey bars) and Shannon entropy (black points, smoothed entropy as red line) for aligned rDNA regions. Similar to Figure 3 in the main text, for each genome, a gene neighboring the first rDNA operon was identified, and used to extract homologous rDNA operons from up to 25 other isolates at the species level. In most cases, the entropy is lower in homologous rDNAs than across all the rDNAs in a given genome. For strains with a low number of complete genomes for comparison available, entropy may be artificially increased (see *Mycoplasma hominis*) or decreased (*Helicobacter cinaedi*). A baseline entropy of greater than 0 may indicate equal distribution of two alleles of the operon either within a genome or across genomes.

Excluding GAGE-B HiSeq *B. cereus*

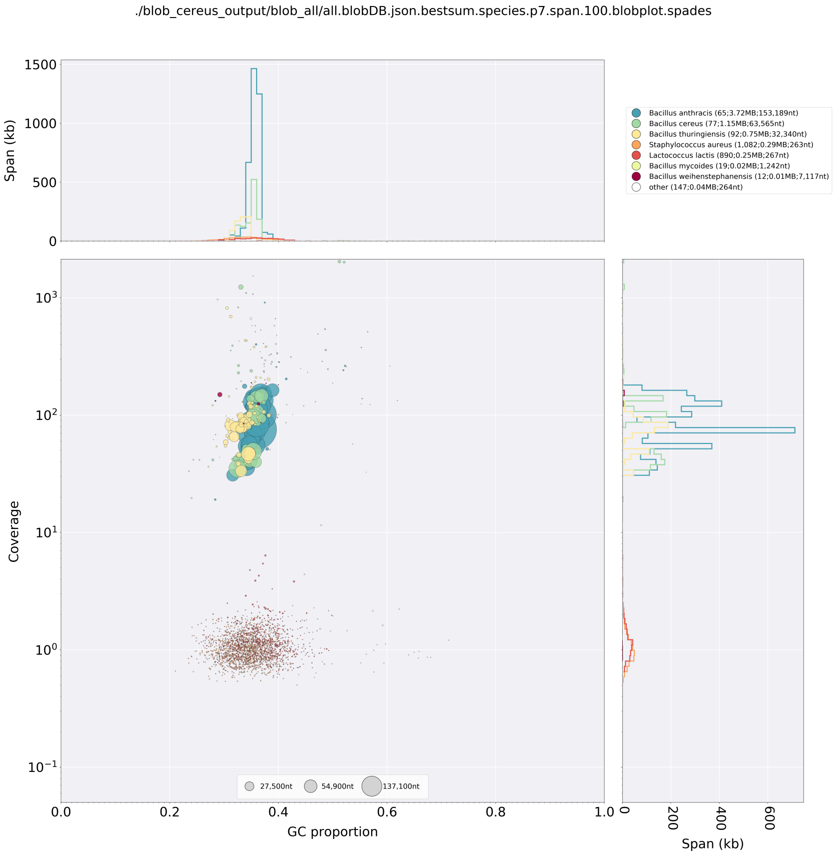

(S5.1) All reads

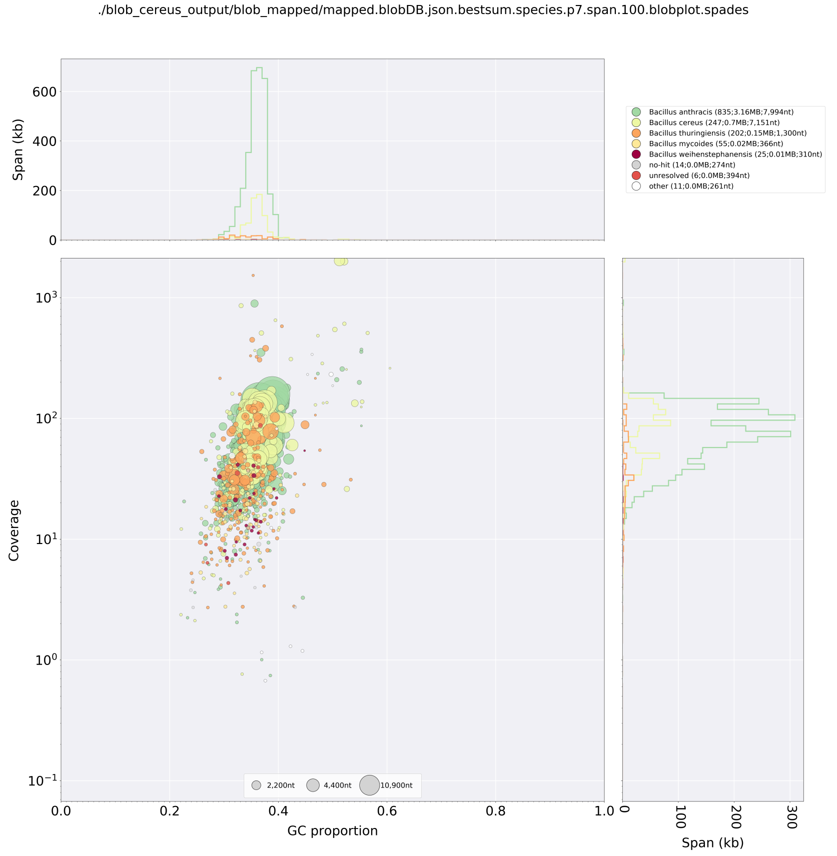

(S5.2) Reads aligning to reference

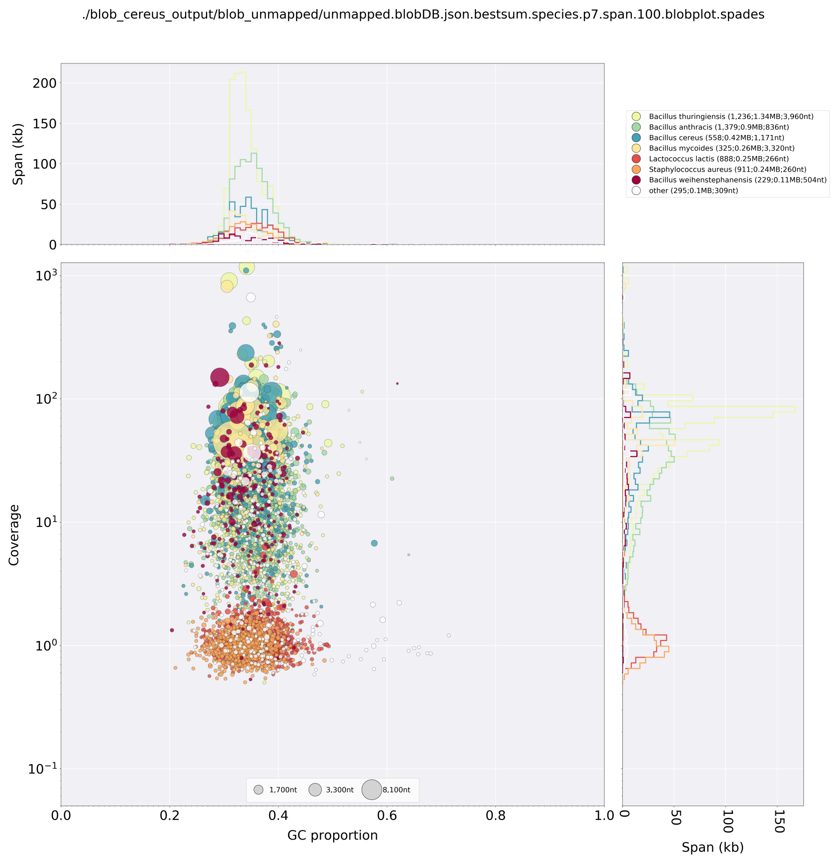

(S5.3) Reads failing to align to reference

Figure S5: Assessing contamination in the GAGE-B HiSeq *B. cereus* dataset with blobtools. Reads were assembled with metaSPAdes, taxanomically assigned with BLASTn against the nt database, and plotted with blobtools. (A) shows the whole dataset, while (B) and (C) shows the portion of the reads aligning to the *B. cereus* ATCC 10987 reference and those failing to align, respectively.

The GAGE-B paper [7] notes that the *B. cereus* HiSeq dataset proved particularly difficult to assemble. After noticing this irregularity, we re-assembled the trimmed reads downloaded from the GAGE-B website with metaSPAdes [8] using default parameters. Then, blastn was used to search the resulting contigs against NCBI's nt database (May, 2017) to get a list of hits according to the blobtools [9] specifications. Blobtools was then used to plot the hit coverage, taxonomy, and GC-content of the contigs. This revealed what appears to be a contamination. S5A. As the GC content of the contaminating organisms did not differ from *B. cereus*, we believe that many tools that use GC-skew to detect contamination would not have detected the problem with this dataset.

To further show the contamination, we split reads into those read pairs mapping to the *B. cereus* ATCC 10987 reference genome and those unmapped. BWA-MEM `http://bio-bwa.sourceforge.net/` was used to map the 12039737 reads to the reference genome; samtools was used to separate the 7500534 reads (62%) that mapped from the 3984200 reads (33%) that failed to map with default parameters[1].Each of these sets of reads was then assembled, BLASTed against the nt database, and plotted with blobtools S5B and C.

Further, MaxBin [10], Kraken, and MBBC [11] also supported the hypothesis that the sample is contaminated with approximately one third of reads originating from a non-*B. cereus* strain.

[1] The remaining ~5 % are those pairs where only one read aligned to the reference; these were ignored for this analysis.

Atypical rDNA operon structure

Bacterial (and many archaeal) ribosomal RNA coding regions are commonly arranged into operons consisting of a 16S rRNA, 23S rRNA, and one or more 5S rRNAs, often with various tRNAs interspersed. In the course of this study, we observed some taxa lacking this typical 16S–23S–5S rRNA operon. When rDNAs are not structured into operons, assemblies from short reads do not suffer from the issue of long repeats and so do not require specialised approaches to assembly, such as riboSeed. We developed a module called `structure` for plotting rDNAs across a collection of genomes; this is available for riboSeed as of version 0.4.50. Figure S6 show the operon arrangement of a few examples of organisms exhibiting atypical operon structure. For comparison, the rDNAs in the reference strains used in this study are shown in Figure S7.

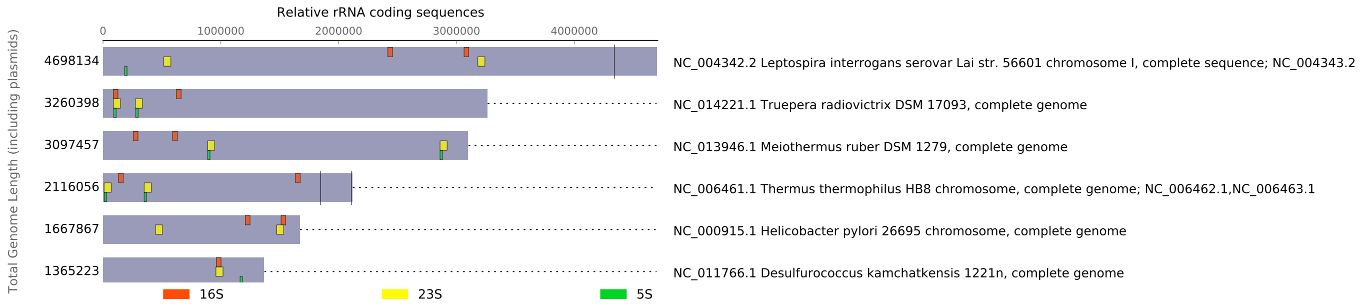

Figure S6: Atypical rDNA operon structure in select taxa. rRNA lengths are not shown to scale. Note that the NCBI record for *Helicobacter pylori* (NC_000915.1) shows a 5S rRNA not detected by Barrnap.

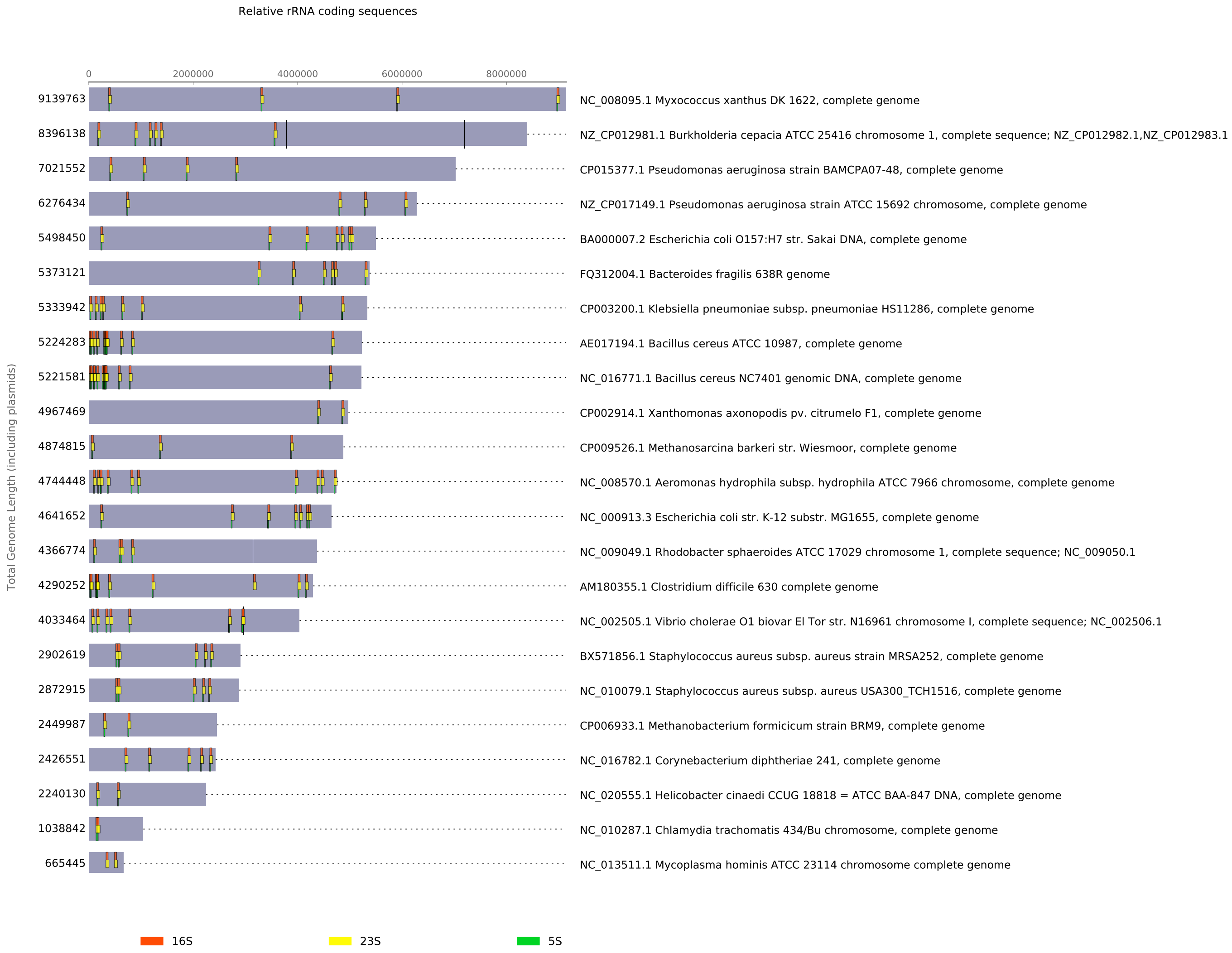

Figure S7: Typical rDNA structure exhibited by strains used in this study.

References

[1] Derrick E Wood and Steven L Salzberg. Kraken: ultrafast metagenomic sequence classification using exact alignments. *Genome Biology*, 15(R46), 2014.

[2] Dhany Saputra, Simon Rasmussen, Mette V. Larsen, Nizar Haddad, Maria Maddalena Sperotto, Frank M. Aarestrup, Ole Lund, and Thomas Sicheritz-Pontén. Reads2Type: a web application for rapid microbial taxonomy identification. *BMC Bioinformatics*, 16(1):398, dec 2015.

[3] Anton Bankevich, Sergey Nurk, Dmitry Antipov, Alexey A Gurevich, Mikhail Dvorkin, Alexander S Kulikov, Valery M Lesin, Sergey I Nikolenko, Son Pham, Andrey D Prjibelski, Alexey V Pyshkin, Alexander V Sirotkin, Nikolay Vyahhi, Glenn Tesler, Max A Alekseyev, and Pavel A Pevzner. SPAdes: A New Genome Assembly Algorithm and Its Applications to Single-Cell Sequencing. *Journal of Computational Biology*, 19(5):455–477, 2012.

[4] Alexey Gurevich, Vladislav Saveliev, Nikolay Vyahhi, and Glenn Tesler. QUAST: quality assessment tool for genome assemblies. *Bioinformatics*, 29(8):1072–1075, apr 2013.

[5] Bruce J. Walker, Thomas Abeel, Terrance Shea, Margaret Priest, Amr Abouelliel, Sharadha Sakthikumar, Christina A. Cuomo, Qiandong Zeng, Jennifer Wortman, Sarah K. Young, and Ashlee M. Earl. Pilon: An Integrated Tool for Comprehensive Microbial Variant Detection and Genome Assembly Improvement. *PLoS ONE*, 9(11):e112963, nov 2014.

[6] Marten Boetzer and Walter Pirovano. Toward almost closed genomes with GapFiller. *Genome Biology*, 13(6), 2012.

[7] Tanja Magoc, Stephan Pabinger, Stefan Canzar, Xinyue Liu, Qi Su, Daniela Puiu, Luke J. Tallon, and Steven L. Salzberg. GAGE-B: an evaluation of genome assemblers for bacterial organisms. *Bioinformatics*, 29(14):1718–1725, 2013.

[8] Sergey Nurk, Dmitry Meleshko, Anton Korobeynikov, and Pavel A. Pevzner. metaSPAdes: a new versatile metagenomic assembler. *Genome Research*, 27(5):824–834, may 2017.

[9] Dominik R. Laetsch and Mark L. Blaxter. BlobTools: Interrogation of genome assemblies. *F1000Research*, 6:1287, jul 2017.

[10] Yu-Wei Wu, Yung-Hsu Tang, Susannah G Tringe, Blake A Simmons, and Steven W Singer. MaxBin: an automated binning method to recover individual genomes from metagenomes using an expectation-maximization algorithm. *Microbiome*, 2(26), 2014.

[11] Ying Wang, Haiyan Hu, and Xiaoman Li. MBBC: an efficient approach for metagenomic binning based on clustering. *BMC Bioinformatics*, 16(36), 2011.
